# Degradable Magnesium Implants with Caerin 1.9-Polycaprolactone Coatings Provide Extended Antibacterial Resistance and Outstanding Biocompatibility

**DOI:** 10.34133/bmr.0257

**Published:** 2025-10-28

**Authors:** Xiaosong Liu, Guoying Ni, Guoqiang Chen, Xiaohong He, Pingping Zhang, Yuandong Luo, Quanlan Fu, Junjie Li, Shuxian Tang, Guowei Ni, Zhengyi Jiang, Tsuyoshi Furushima, Damon Kent, Bin Zhu, Tianfang Wang, Hejie Li

**Affiliations:** ^1^ Cancer Research Institute, First People’s Hospital of Foshan, Foshan, Guangdong 528000, China.; ^2^ The First Affiliated Hospital, Clinical Medical School, Guangdong Pharmaceutical University, Guangzhou 510080, China.; ^3^ Centre for Bioinnovation, University of the Sunshine Coast, Maroochydore BC, QLD 4558, Australia.; ^4^ Department of Rheumatology, First People’s Hospital of Foshan, Foshan, Guangdong 528000, China.; ^5^ Department of Medical Imaging, First People’s Hospital of Foshan, Foshan, Guangdong 528000, China.; ^6^ School of Civil Architectural Engineering, North China University of Science and Technology, Tangshan 063000, China.; ^7^ School of Mechanical, Materials, Mechatronic and Biomedical Engineering, Faculty of Engineering and Information Sciences, University of Wollongong, Wollongong, NSW 2522, Australia.; ^8^ Institute of Industrial Science, Department of Mechanical and Biofunctional Systems, the University of Tokyo, Meguro, Tokyo 153-8505, Japan.; ^9^ School of Science, Technology and Engineering, University of the Sunshine Coast, Maroochydore BC, QLD 4558, Australia.

## Abstract

Implant-associated infections and foreign body responses remain major challenges in orthopedic and biomedical implant applications. In this study, we report a novel strategy to enhance the antibacterial and biocompatibility properties of magnesium (Mg) alloy implants by applying a biodegradable polycaprolactone (PCL) coating embedded with the host-defense peptide, caerin 1.9 (F3). Three Mg-based specimens, including pure Mg, cold-extruded AZ31, and fully annealed AZ31 (3A), were evaluated following PCL-F3 surface modification. The PCL-F3 coatings demonstrated sustained antibacterial efficacy both in vitro and in vivo, effectively inhibiting methicillin-resistant *Staphylococcus aureus* (MRSA) for up to 168 h. Among the groups, the 3A-PCL-F3 condition exhibited the most notable performance, with substantially enhanced corrosion resistance, reduced inflammatory responses, and no detectable toxicity to vital organs. In vivo proteomic and metabolomic analyses further revealed that the 3A-PCL-F3 implants promoted the expression of osteogenic markers and activated pathways related to bone mineralization and hemostasis, while avoiding prolonged inflammatory activation at 3 months post-implantation. Notably, histological and cytokine ELISA data confirmed favorable tissue responses, including suppressed IL-1β and IL-10 levels and signs of early immune activation that subsided over time. These findings indicate that PCL-F3-coated Mg alloys, particularly the 3A variant, represent a promising solution for biodegradable implants with dual antibacterial and regenerative functionality. This work lays the foundation for developing degradable Mg alloy biomaterials with enhanced biocompatibility and multifunction for clinical use.

## Introduction

Globally, populations are aging, presenting ‌enormous challenges to healthcare systems worldwide. Among the pressing issues associated with aging are fractures resulting from accidents and osteoporosis, particularly affecting the elderly [1]. Studies reveal that around half of women and one-fifth of men aged 50 and above have encountered at least one fracture [2–4]. Conventional bone internal fixation materials, such as titanium and stainless-steel alloys, exhibit notable differences in mechanical and physical properties compared to human bone tissue with negative impacts on biocompatibility. In particular, notable differences in elastic moduli cause “stress shielding” around the implant leading to failure [5]. Additionally, local release of metal ions elevates pH levels and increases risk of infection or inflammatory reactions [6]. Conversely, polymer implant materials face limitations due to their inferior mechanical properties [7]. Whether used as permanent or temporary implants, these materials often necessitate multiple surgeries, leading to unfavorable hospital experiences and financial burdens for patients [2]. Thus, there is a growing demand for materials with better biocompatibility for internal fixation, repair, and replacement of bone tissue. Among them, degradable metal-based biomaterials with compositions based on nutritionally essential trace elements (Mg, Fe, and Zn) are receiving substantial attention as they can provide necessary mechanical support and then degrade naturally with outstanding biocompatibility [8].

Orthopedic applications impose stringent requirements on implants, encompassing mechanical and corrosion properties. These criteria include excellent mechanical strength (elastic modulus: 10 to 20 GPa), osseointegration capability, and outstanding wear and corrosion resistance and/or degradation products that are well-tolerated in the human body. Magnesium (Mg) is emerging as a promising solution as it possesses a density and modulus akin to human bones [9]. Mg and its alloys provide high specific strength and the appropriate stiffness needed for hard tissue implants [10]. Moreover, Mg is essential for human health, playing a pivotal role in numerous physiological processes. Adults typically intake Mg^2+^ daily in the range of 240 to 420 mg, greatly surpassing intakes of other beneficial elements such as Fe^3+^ (8 to 18 mg) and Zn^2+^ (8 to 11 mg) [11]. Over 60% of Mg in the human body is stored in the bones and muscles, totaling around 30 g [12]. Mg participates in various metabolic processes, including protein synthesis, enzyme activation, regulation of the central nervous system, muscle function, and the operation of vital organs like the intestines and stomach. Additionally, Mg engages in physiological activities like calcium antagonism and serves as a signal transmitter [13]. Despite its advantageous properties, the rapid degradation of Mg alloys in the human body has limited their clinical use, and many approaches, including alloying [2,14,15], processing, and surface modification [16,17], have been used to improve the corrosion resistance of Mg alloys. These techniques are also popularly employed in the protective coatings [18–21].

Another noticeable issue adversely affecting the usage of metal-based implant materials is periprosthetic infection (PPI), resulting from bacterial accumulation, colonization, and biofilm formation [19,22]. Clinical treatments for PPI involve antimicrobial therapies, surgical interventions, and implant removal and replacement, all of which necessitate periods of post-operative recovery. This often leads to physical and mental discomfort for patients, along with unexpected expenses [23]. Recent studies have revealed that pure Mg exhibits antibacterial behaviors in both in vitro and in vivo settings [9]. However, its rapid degradation can lead to adverse physiological effects, including alkalosis, local inflammation, and cell death [19]. Consequently, Mg alloys in conjunction with surface engineering, which slows degradation and enhances antibacterial effects, show promise for the next generation of degradable Mg alloy-based biomaterials.

Previously, we developed a chemical click-based polyurethane (PU) coating to improve corrosion resistance and biocompatibility of Mg alloys [2]. The coating incorporated one type of natural cationic host-defense peptides (HDPs), caerin 1.9, which conferred potent antibacterial properties to the Mg alloy surfaces. HDPs, also known as antimicrobial peptides, are evolutionarily conserved molecules widely expressed across diverse species. They exert antimicrobial effects through direct microbicidal activity and/or by modulating host immune responses, including inflammation suppression [24].

Caerin 1.9, a peptide derived from the Australian tree frog (*Litoria* genus) [25], has emerged as a particularly promising candidate for biomedical applications due to its dual antibacterial [2–5] and immunomodulatory functions [6–12]. Caerin 1.9 has been shown to disrupt bacterial membranes through electrostatic interaction and pore formation, leading to rapid bacterial cell death [3,4]. In addition to its direct bactericidal effects, caerin 1.9 also inhibits biofilm formation and has demonstrated activity against multidrug-resistant strains, including methicillin-resistant *Staphylococcus aureus* (MRSA). Beyond its antimicrobial capabilities, recent studies have highlighted its role in modulating immune responses and inflammation, which are critical in promoting tissue repair and reducing foreign body reactions associated with implanted materials.

In our earlier work, we immobilized caerin 1.9 onto variously prepared Mg alloy substrates [2]. Notably, the caerin-coated and fully annealed AZ31 Mg alloy (Mg-3Al-1Zn wt.%) showed greatly improved corrosion resistance with sustained in vitro antibacterial activity lasting up to 120 h, which is considerably longer than conventional antibiotic-based treatments [2]. Furthermore, the fully annealed AZ31 alloy displayed strong in vivo antibacterial resistance and facilitated tissue healing by activating signaling pathways involved in regeneration and immune modulation [3].

In this study, we developed a novel 3-dimensional (3D) coating system using the biodegradable polymer polycaprolactone (PCL), incorporating caerin 1.9 to further enhance the antibacterial efficacy of Mg alloy implants. The use of bulkier 3D coatings allows for a prolonged degradation profile and sustained release of the bioactive peptide into the local microenvironment, offering more durable antibacterial effects compared to previous 2-dimensional (2D) PU systems. We systematically evaluated the in vitro and in vivo responses of coated pure Mg (2P), cold-extruded AZ31 (1E), and fully annealed AZ31 (3A) alloys, focusing on antibacterial performance against MRSA in a rat subcutaneous implantation model.

## Materials and Methods

### Mg alloys and specimen preparation

Three different Mg alloys were used in this study, including 1E, 2P, and 3A. The 3A samples underwent a full recrystallization annealing heat treatment, as described previously [2,3]. In brief, they were heated to 330 to 350 °C in argon, held for 3 to 5 h, and then furnace cooled. The specimens were fabricated as small pins with a thickness of 5 and 2 mm. Before the in vitro and in vivo experiments, the alloy samples were polished. They were initially treated with 400-grit silicon carbide paper for 1 to 3 min to remove the original oxide layer. Then, they were polished by 800- to 2,400-grit silicon carbide paper for 2 to 5 min to improve the sample surface qualities and achieve uniform roughness. After each step of polishing and grinding, the specimens were rotated by 90° to ensure that the subsequent procedures removed the scratches generated in the previous step. Finally, all samples were cleaned in 70% ethanol at room temperature for 5 min using ultrasonics.

### Peptide synthesis

Caerin 1.9 (F3) (GLFGVLGSIAKHVLPHVVPVIAEKL-NH_2_) was synthesized by ChinaPeptides Co., Ltd. (Shanghai, China). The purity of the peptides exceeded 99% as determined by ChinaPeptides Co., Ltd. using reverse-phase high-performance liquid chromatography [2].

### Cell lines and osteoblast adhesion test

MC3T3-E1 cells were procured from the cell resource center of Shanghai Institutes of Biological Sciences, Chinese Academy of Sciences. MC3T3-E1 osteoblasts were seeded into a 6-well plate with 1.0 × 10^6^ cells and 2 ml of culture medium per well. All the specimens were divided into 4 groups: 1 group is untreated, and the 3 other groups are Ti, 316L, and 3A, respectively. Three metal specimens were placed into 6-well plates according to group set and cultured together for 48 h. After 48 h, the supernatant was collected completely, then 300 μl of trypsin was added, and the solution was placed in a cell culture incubator to digest the metal-treated MC3T3-E1 cells for 2 min. After 2 min, the cells were taken out, then observed under a microscope for the analysis of cells’ digestion, and photos were taken. The undigested MC3T3-E1 cells were placed in the incubator to continue the digestion until the cells were fully digested.

### EBSD mapping

Electron backscatter diffraction (EBSD) mapping of the Mg alloy microstructures was performed at the Institute of Industrial Sciences of the University of Tokyo using a JOEL JSM-7100F field emission gun scanning electron microscope. The EBSD scanning was carried out with an accelerating voltage of 15 kV and a scanning step size of 0.5 μm. Further details on the EBSD sample preparation method can be found in Ref. [3]. The EBSD mapping position was as follows: ED/RD refers to the extrusion/rolling direction, TD is the transverse direction, and ND is the normal direction. The analysis of the EBSD mapping results was conducted using orientation-imaging microscopy V7.0.

### Microhardness test

Microhardness tests were performed on the round specimens (diameter is 5 mm and thickness is 2 mm) with a roughness of 800 using an HMV-G micro-Vickers hardness tester at the Institute of Industrial Sciences of the University of Tokyo, applying a load of HV0.01 (98.07 mN) [2,3].

### Uniaxial tensile test

Uniaxial tensile tests were performed using a 100 KN Shimadzu universal material testing machine at the University of the Sunshine Coast. The Mg sample had the following dimensions: an engaged length of 30 mm, an overall length of 110 mm, and a rod diameter of 5 mm.

### Physical 3D coating of PCL and caerin peptide F3

**Fig. 1.**
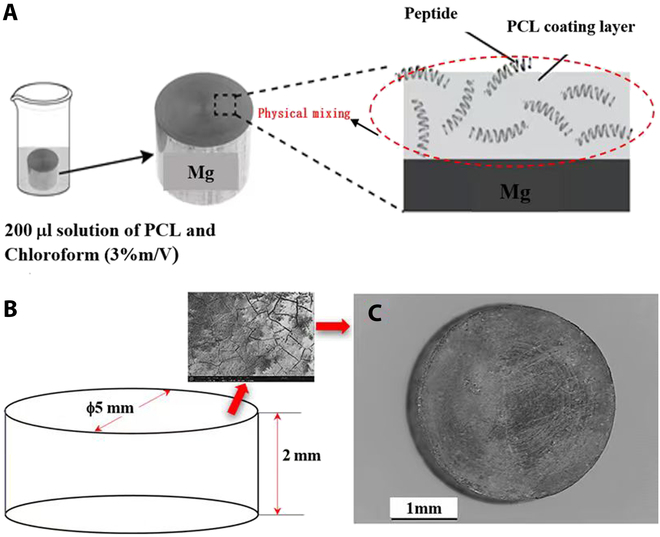
(A) Schematic of the coating method. (B) Specimen’s size and surface (FIB-SEM). (C) PCL-F3-coated Mg alloy.

The samples prepared following the “Mg alloys and specimen preparation” section were inactivated by exposing to ultraviolet (UV) light for 30 min prior to the coating process (Fig. 1A). Biocompatible PCL was chosen as the coating material for the metal surface. PCL pellets were dissolved in 10 ml of pure chloroform to a ratio of 2% W/V, and then 45 mg of F3 was added to the chloroform-PCL solution, followed by thorough vortex mixing at room temperature for approximately 2 h until a homogeneous solution was achieved. The metal samples were immersed in the solution and air-dried in a fume hood for 10 min. This step was repeated 4 more times. Figure 1B and C provide visual representations of the Mg alloy samples with F3 coating.

### FTIR test

Infrared (IR) spectra were obtained on a Spectrum Two FTIR (Fourier transform infrared) spectrometer (Perkin-Elmer, Waltham, MA) at room temperature, with the scan wavelength 4,000 to 5,500 cm^−1^. As a comparison, the IR spectra of PCL coating (without peptide immobilization) and PCL-F3 coating were also measured.

### Water contact angle test

Water contact angle measurements were conducted using a drop shape analyzing by dropping with pure water to evaluate the hydrophilicity of the different titanium surfaces [26].

### In vitro antibacterial test

The in vitro antibacterial tests were conducted by following our previous method [2]. After 3 to 5 min of ultrasonic cleaning in distilled water, all F3- and PCL-coated Mg alloy samples (1E-PCL-F3, 2P-PCL-F3, and 3A-PCL-F3) were put into a bacteriostatic petri dish to conduct a 100-h bacteriostatic test on drug-resistant *S. aureus* in a 37 °C temperature incubator. The MRSA (GDM1.1263) were cultured to a logarithmic phase, and the suspension concentration of the MH (Mueller–Hinton) medium was adjusted to 2.0 × 10^5^ colony-forming units/ml. A sterile cotton swab was used to dip the bacteria solution and squeeze the tube wall several times to remove excess. The swab was used to smear the entire M-H drug-sensitive agar plate (Guangzhou Yuanming Bio Company). Aliquots of 30 μg of F1 and F3 peptides were added to drug-sensitive papers (OXOID, UK), and the papers were pasted on M-H agar plates. The plates were inverted and incubated at 37 °C overnight. A volume of 30 μg of piperacillin sodium and tazobactam sodium with a weight ratio of 8:1 (Tazocin, Haikou Qili Pharmaceutical Co., Ltd, Haikou) and blank drug-sensitive tablets (BASD, Thermo Fisher Scientific, Shanghai) and 2 original AZ31 Mg alloys were used as controls. A Vernier caliper was used to measure the size of the zone of inhibition.

### HE staining

Place all tissues in numbered test tubes, add 10% formalin solution to fix the tissues, and perform hematoxylin–eosin (HE) staining after fixing the tissues for 24 h. The specific steps are as follows: (a) Sample tissue fixation and sectioning: embed tissue samples in conventional paraffin. Add liquid paraffin to the mold and place the tissue samples to be embedded in the paraffin to ensure that the tissues are in a regular position. After adding a small amount of liquid paraffin, cool and freeze the paraffin to solidify the tissue to achieve the effect of tissue fixation and then use a paraffin slicer to slice the tissues with a thickness of about 4 to 8 μm. Place the cut tissue slices on a slide and soak them in 40 °C warm waters to fully stretch the tissues. (b) Tissue sample dewaxing: Place the tissue sample slices to be tested in xylene and soak them for 10 min. After soaking, replace xylene and continue soaking for 10 min to fully dissolve the paraffin in the slices. (c) Hydration of tissue samples: Soak the tissue samples soaked in xylene in anhydrous ethanol for 5 min to allow the xylene used in dewaxing to be washed out and water to enter the tissue; then soak them in 95%, 85%, and 70% ethanol for 5 min each to achieve full hydration. (d) Hematoxylin staining, differentiation, and anti-bluing of tissue sections: Soak the sections of the hydrated tissue samples in phosphate-buffered saline solution, use a pipette to absorb the hematoxylin staining solution, add 100 μl to each tissue section, and fully stain for 10 min. After staining, use distilled water to wash away the excess hematoxylin staining solution. Use 1% hydrochloric acid ethanol to remove the excess staining solution and rinse the tissue sections. (e) Eosin staining and dehydration of tissue sections: Add eosin staining solution to allow the tissue to be fully stained for 3 min. After staining, dehydrate the tissue sections in a gradient manner, using 80%, 95%, and anhydrous ethanol, respectively. Dehydrate with 80% ethanol for 5 s, 95% ethanol for 2 min, and anhydrous ethanol for 2 min. (f) Air-drying and sealing of tissue sample slices: Soak the dehydrated tissue sample slices in xylene twice, each time for 4 min, then air-dry the tissue sample slices and seal them with neutral gum.

### MTT test

MC3T3-E1 cells were seeded in 96-well plates (1.0 × 10^4^ cells per well, 0.1 ml medium) overnight and divided into 4 groups, which were the control group and the 1E, 2P, and 3A metal material groups. On the second day, 3 kinds of metal materials, 1E, 2P, and 3A, were added to the 96-well plates, respectively, cultured in the incubator for 24 h, and the metal materials were removed. Five milligrams per milliliter of 20 μl of 3-(4,5-dimethylthiazol-2-yl)-2,5-diphenyltetrazolium bromide (MTT) was added to each well and incubated in a 5% CO_2_ incubator at 37 °C for 4 h. The culture medium was removed, 150 μl of dimethyl sulfoxide was added, and an optical density value of 490 nm absorbance was measured by Microplate Reader (Thermo Fisher Scientific). Ordinary one-way analysis of variance (ANOVA) with Tukey’s post-hoc test was employed, with the results displayed in Table S1.

### Real-time PCR test

#### RNA isolation

After co-culturing osteoblasts with metals 3A, Ti, and 316L for 48 h, the metals were removed. The cells were then treated with trypsin, followed by centrifugation at 1,200 rpm for 5 min. RNA from the osteoblasts was extracted using the Trizol method. The quality and concentration of the isolated RNA samples were evaluated using a micro-UV-Vis spectrophotometer (AmoyDxNanoDrop 2000c, Xiamen, China).

#### Quantitative PCR

The expressions of BRF1, Ctnna1, and KAT6A genes in osteoblasts were detected using the quantitative PCR (qPCR) method. A β-actin-specific primer was used as an internal control. The sequences of the gene primers used for amplification are listed in Table S2. cDNA was synthesized from 1 μg of RNA using the PrimeScript RT reagent Kit with gDNA Eraser (Perfect Real Time). qPCR was performed according to the instructions of the TB Green Premix Ex Taq II (Tli RNaseH Plus) (TaKaRa RR820A). The instrument used was the Roche LC480 from Roche Diagnostics, Switzerland. The thermal cycling conditions were set as follows: initial denaturation at 95 °C for 30 s, followed by 40 cycles of PCR reaction: 95 °C for 5 s and 60 °C for 20 s, and a dissociation curve analysis: 95 °C for 5 s, 60 °C for 1 min, and 95 °C for 0 s. Each qPCR was conducted in triplicate. Ordinary one-way ANOVA with Tukey’s post-hoc test was employed, with the results recorded in Table S1.

### In vivo test

#### Rats

Six- to eight-week-old Sprague–Dawley (SD) rats were procured from the Animal Resource Centre of Guangdong Province. The rats were housed at the Animal Facility of the Foshan First People’s Hospital in Guangdong, China. All experimental procedures were approved and conducted in accordance with the guidelines of the Animal Experimentation Ethics Committee (Ethics Approval Number: C202307-5) by the Foshan First People’s Hospital, the University of the Sunshine Coast’s Animal Ethics Committee (Ethics Approval Number: ANE23105). The rats were maintained in specific-pathogen-free (SPF) conditions on a 12-h light/dark cycle at 22 °C with 75% humidity. Each rat was individually housed in a cage and provided with sterilized standard mouse food and water. At the conclusion of each experiment, rats were euthanized by CO_2_ inhalation, confirmed by the cessation of breath and heart function [2,21]. For all the tests, the number of animals per group is 5.

#### Metal implants in rat femur

The ultrasonically cleaned specimens were exposed to UV radiation for 30 min on each side for sterilization [2]. The implantation was conducted in the animal house of Foshan First People’s Hospital. Twelve 8-week-old male SPF SD rats were weighed at 266.646 g. Rats were randomly divided into 4 groups, including control (no implants), 1E, 2P, and 3A groups. Rats were anesthetized by intraperitoneal injection of 1% sodium pentobarbital solution with a dose of 40 mg/kg. A sterile blade was used to cut about 1 cm perpendicular to the femoral shaft, then the subcutaneous tissue and muscle were separated until the femoral condyle of the rats was exposed. A grinding drill was used to drill a hole located at the lateral condyle of the rats’ femur perpendicular to the longitudinal axis of the femur [3]. The control group was not embedded with any implant after drilling. The incision was sutured layer by layer with 4-0 absorbable sutures.

#### Degradation and biocompatibility of implants

After 3 days of implantation, the rats were anesthetized via intraperitoneal injection of 1% sodium pentobarbital at the dose of 40 mg/kg. Peripheral blood samples were collected by eye bleeding for the investigation of serum electrolyte, liver, and kidney function. Following the blood collection, the rats were euthanized. The implants were removed from the femoral condyle, cleaned, dried, and disinfected. After the complete removal of attached soft tissue was completely removed, the implants were photographed to evaluate the degree of degradation. The organ tissues (including heart, liver, spleen, lung, kidney, brain, ovary, etc.) were collected and fixed with 10% formalin for HE staining. Furthermore, several other groups’ tests last about 3 months. Post 3 months, the same tests including degradation and biocompatibility will be repeated.

### SEM-EDS analysis

Mg alloy specimens were removed from the SD rats after 3 days of implantation, then cleaned ultrasonically with 70% ethanol and rinsed in distilled water for 3 to 5 min. Scanning electron microscopy–energy dispersive x-ray spectroscopy (SEM-EDS) analysis was conducted on Zeiss Sigma 300 Field Emission Electron Gun (FEG)-SEM with the following parameters: extra high tension is 3.00 kV, working distance is 5.4, and magnification is 100×.

### Computerized tomography imaging

Three-dimensional computerized tomography (3D CT) scans were conducted at the First People’s Hospital of Foshan. A clinical 64 slices CT system, specifically the GE Discovery 64 model from GE Healthcare (Waukesha, USA), was utilized. All 3D CT imaging was performed while the rats were under anesthesia [3]. The results were not listed in this study because they are not that accurate.

### Cytokine ELISA

Cytokine ELISA for rat sera, targeting tumor necrosis factor α (TNFα), interleukin-10 (IL-10), monocyte chemoattractant protein-1 (MCP-1), and IL-1β, was conducted using kits obtained from R&D Systems (Minneapolis, USA). The assays were performed following the manufacturer’s protocol provided.

### Proteomics analysis

#### Protein sample preparation

Either MC3T3-E1 cells collected from the co-culture with Mg specimen or mouse tissue samples were homogenized in SDT buffer (4% SDS, 100 mM Tris-HCl, and 1 mM DTT, pH 7.6), and 200 μg of proteins for each sample was subjected to trypsin digestion according to the filter-aided sample preparation procedure described elsewhere [3]. The protein suspensions were digested with trypsin (Promega) overnight at 37 °C, and the resulting peptides were desalted on C18 Cartridges (Empore SPE Cartridges C18, bed I.D. 7 mm, volume 3 ml, Sigma), and lyophilized by vacuum centrifugation for TMT10plex labeling. A total of 100 μg of peptide mixture of each sample was labeled using TMT reagent according to the manufacturer’s instructions (Thermo Fisher Scientific). Labeled peptides were fractionated by SCX chromatography using an AKTA Purifier system (GE Healthcare). The collected fractions were desalted on C18 Cartridges, lyophilized, and resuspended for LC-MS/MS analysis (see Supplementary Methods for more details).

#### NanoLC tandem Q-Exactive MS/MS analyses

The peptide samples were analyzed using a Q Exactive mass spectrometer coupled to Easy nLC (Thermo Fisher Scientific) following the method detailed previously. In brief, the peptides were loaded onto a reverse-phase trap column (Thermo Fisher Scientific Acclaim PepMap100, 100 μm × 2 cm, nanoViper C18) connected to the C18 reverse-phase analytical column (Thermo Fisher Scientific Easy Column, 10 cm long, 75 μm inner diameter, 3 μm resin) in buffer A (0.1% formic acid) and separated with a linear gradient of buffer B (84% acetonitrile and 0.1% formic acid) at a flow rate of 300 nl/min. The mass spectrometer was operated in positive ion mode. MS data were acquired using a data-dependent top 10 method dynamically selecting the most abundant precursor ions from the survey scan (300 to 1,800 *m*/*z*) for higher-energy collisional dissociation fragmentation.

#### Protein identification and quantitation

The MS/MS data were searched against Ensembl_Rattus_29107_20200311 (76,417 sequences, downloaded on 2014 December 12) database for protein identification using Mascot2.2 (Matrix Science, London, UK) and Proteome Discoverer1.4 software (Thermo Fisher Scientific, Waltham, MA, USA) with the following search settings: enzyme trypsin; 2 missed cleavage sites; precursor mass tolerance 20 ppm; fragment mass tolerance 0.1 Da; fixed modifications: carbamidomethyl (C), TMT 10plex (N-term), TMT10 plex (K); variable modifications: oxidation (M), TMT 10plex (Y). The results of the search were further submitted to generate the final report using a cutoff of 1% FDR on peptide levels and only unique peptides were used for protein quantitation. All peptide ratios were normalized by the median protein ratio, and the median protein ratio was 1 after the normalization. The significance of protein contents was statistically analyzed using the Student’s *t* test. The protein shows a fold change ≥ 1.2 compared to the control group, and *P* values < 0.05 were considered significantly regulated.

#### Gene ontology, domain, and KEGG pathway analysis

The protein sequences of differentially expressed proteins were locally searched using the NCBI BLAST+ client software (Version. 2.2.28). Gene ontology (GO) terms were mapped, and sequences were annotated using the OMICSBOX software (https://www.biobam.com/omicsbox/). InterProScan software within OMICSBOX was used to identify protein domain signatures from the InterPro member database Pfam. Protein sequences were also compared with the online Kyoto Encyclopedia of Genes and Genomes (KEGG) database (http://geneontology.org/) to retrieve their KEGG orthology identifications and map them to pathways in KEGG. Enrichment analysis was performed using the Fisher’s exact test, with the entire set of quantified proteins as background dataset. Benjamini–Hochberg correction for multiple testing was applied to adjust derived *P* values. Only functional categories and pathways with *P* value less than 0.05 were considered statistically significant.

#### Protein–protein interaction analysis

The protein–protein interaction (PPI) information for the studied proteins was obtained from the IntAct molecular interaction database [27] (http://www.ebi.ac.uk/intact/) using their gene symbols. Alternatively, STRING (http://string-db.org/) was used for PPI retrieval. The results obtained were downloaded and visualized through Cytoscape software (http://www.cytoscape.org/, version 3.2.1). The statistical analysis of the PPI was conducted using the Network Analyser [28] in Cytoscape.

### Metabolomics analysis

#### Sample preparation and extraction

The rat tissue samples stored in a −80 °C refrigerator was thawed on ice. The thawed sample was homogenized for 20 s using a grinder operating at 30 Hz. A 400-μl solution (methanol:water = 7:3, V/V) containing an internal standard was added to 20 mg of ground sample and shaken at 1,500 rpm for 5 min. After allowing it to cool on ice for 15 min, the sample was centrifuged at 12,000 rpm for 10 min at 4 °C. A 300-μl portion of the supernatant was collected and placed in a −20 °C refrigerator for 30 min. The sample was then centrifuged at 12,000 rpm for 3 min at 4 °C. Aliquots (200 μl) of supernatant were transferred for LC-MS analysis.

#### LC-ESI-MS/MS analysis

The sample extracts were analyzed using an LC-ESI-MS/MS system (UPLC, ExionLC AD; MS, QTRAP). The analytical conditions were as follows: UPLC: column, Waters ACQUITY UPLC HSS T3 C18 (1.8 μm, 2.1 mm × 100 mm); column temperature, 40 °C; flow rate, 0.4 ml/min; injection volume, 2 μl; solvent system, water (0.1% formic acid): acetonitrile (0.1% formic acid); gradient program, 95:5 V/V at 0 min, 10:90 V/V at 11.0 min, 10:90 V/V at 12.0 min, 95:5 V/V at 12.1 min, 95:5 V/V at 14.0 min. Linear ion trap (LIT) and triple quadrupole (QQQ) scans were acquired on a QTRAP LC-MS/MS System, equipped with an ESI Turbo Ion-Spray interface, operating in positive and negative ion mode and controlled by Analyst 1.6.3 software. The ESI source operation parameters were as follows: source temperature, 500 °C; ion spray voltage (IS), 5,500 V (positive), −4,500 V (negative); ion source gas I (GSI), gas II (GSII), and curtain gases (CUR) were set at 55, 60, and 25.0 psi, respectively; the collision gas (CAD) was high. Instrument tuning and mass calibration were performed with 10 and 100 μmol/l polypropylene glycol solutions in QQQ and LIT modes, respectively. A specific set of multiple reaction monitoring transitions were monitored for each period according to the metabolites eluted within this period.

#### Data analysis

Unsupervised PCA (principal component analysis) was performed by the statistics function “prcomp” within R (www.r-project.org). The data were scaled to unit variance before conducting unsupervised PCA. The results of HCA (hierarchical cluster analysis) for samples and metabolites were presented as heatmaps with dendrograms. Pearson correlation coefficients (PCCs) between samples were calculated by the “cor” function in R and displayed as heatmaps. Both HCA and PCC calculations were carried out using the R package ComplexHeatmap. For HCA, normalized signal intensities of metabolites (unit variance scaling) are visualized as a color spectrum.

Noticeably‌ regulated metabolites between groups were determined based on the criteria of variable importance in projection (VIP) ≥1 and an absolute Log2FC (fold change) ≥ 1. VIP values were extracted from orthogonal partial least squares discriminant analysis (OPLS-DA) results, which also included score plots and permutation plots. The data were log-transformed (log2) and mean-centered before OPLS-DA. To prevent overfitting, a permutation test (200 permutations) was performed. Identified metabolites were annotated using the KEGG Compound database, and the annotated metabolites were subsequently mapped to the KEGG Pathway database. Significantly enriched pathways were identified using a hypergeometric test’s *P* value for a given list of metabolites.

## Results

### Characteristics of Mg alloy specimens

**Fig. 2.**
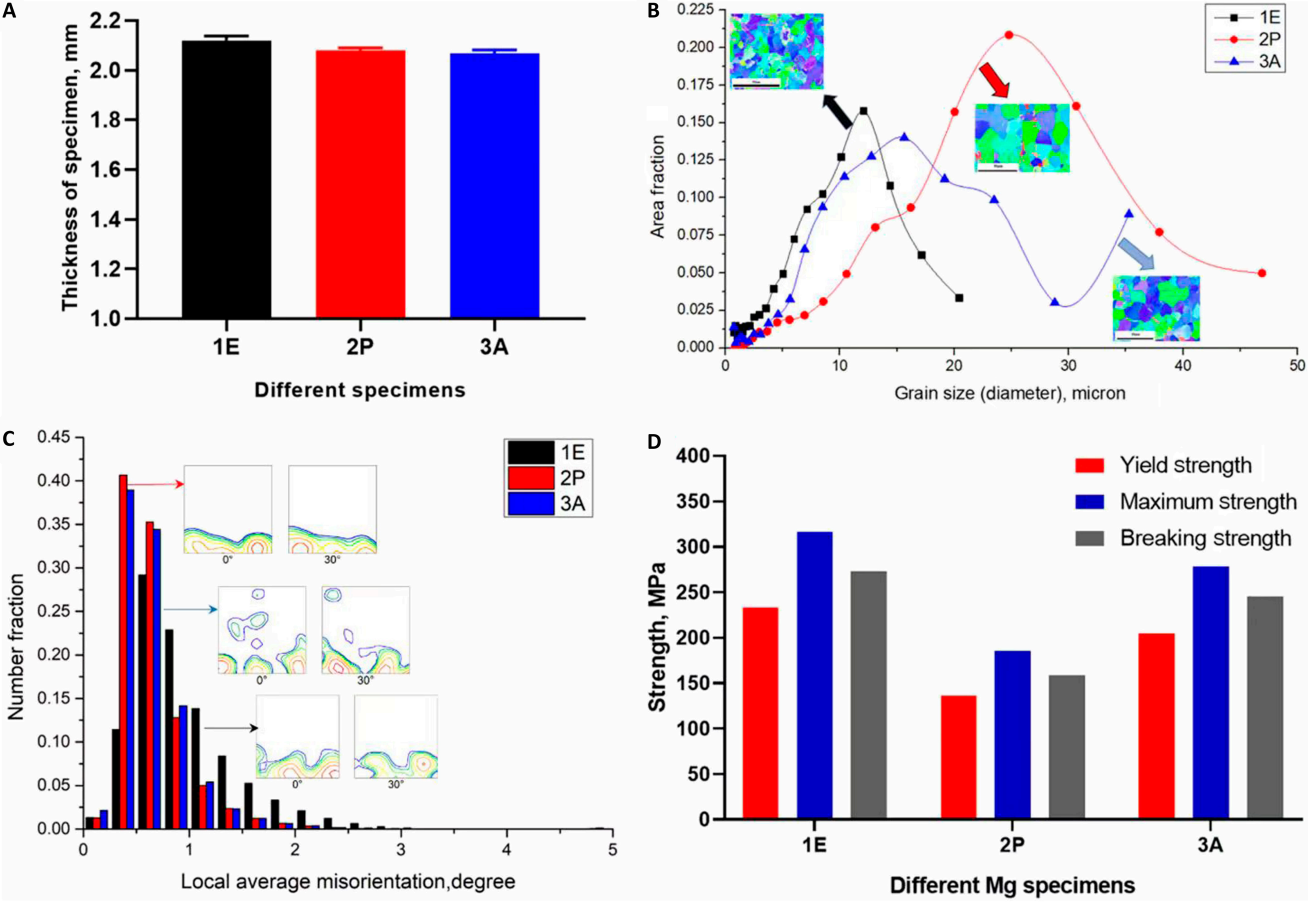
Mg alloy microstructure and mechanical property characterization of different Mg alloy specimen groups. (A) Thickness. (B) Grain size distributions. (C) Local average misorientation angles. (D) Strength under uniaxial tensile test.

The physical characteristics, microstructure, and mechanical properties of the Mg alloy substrates, 1E, 2P, and 3A, were assessed. The average thicknesses of the 3 groups of specimens are presented in Fig. 2A: 2P is thickest at 2.1 mm; 2E and 3A have the similar thickness, with values of 2.07 and 2.05 mm, respectively. The average grain sizes were 22.2 μm for 2P, 9.2 μm for 1E, and 15.9 μm for 3A (Fig. 2B) samples. The grain misorientation angles of 2P and 3A showed similar tendencies, with neighboring grains exhibiting differences mainly below 1°, suggesting that the 2 annealed Mg alloy microstructures have similar characteristics (Fig. 2C). Meanwhile, for 1E, the proportion of grains with higher misorientation angle tends to be greater. Cold extrusion has led to different texture and microstructure along the extrusion direction (extrusion axis), while 2P and 3A both have more homogeneous textures and microstructures.

1E had the highest average microhardness of 81.6 HV, 2P had the lowest average microhardness with a value of 41.5 HV, while 3A had a microhardness of 66.5 HV [2,3]. Uniaxial testing results for the different Mg samples are shown in Fig. 2D. Among the 3 groups, 1E has the highest strength with a yield strength of 233.4 MPa, a maximum ultimate uniaxial tensile strength of 316.1 MPa, and a breaking strength of 272.7 MPa. 3A has the second highest strength with a yield strength of 204.8 MPa, a maximum strength of 277.9 MPa, and a breaking strength of 245.2 MPa. Finally, 2P has the lowest uniaxial tensile strength with a yield strength of 136.2 MPa, a maximum strength of 185.7 MPa, and a breaking strength of 159.1 MPa. Both 1E and 3A exceed strength requirements to be compatible with human bone, which typically has a yield strength of the order of 130 to 180 MPa [29].

### Interaction of uncoated Mg alloy specimens with mouse osteoblast precursor cells

**Fig. 3.**
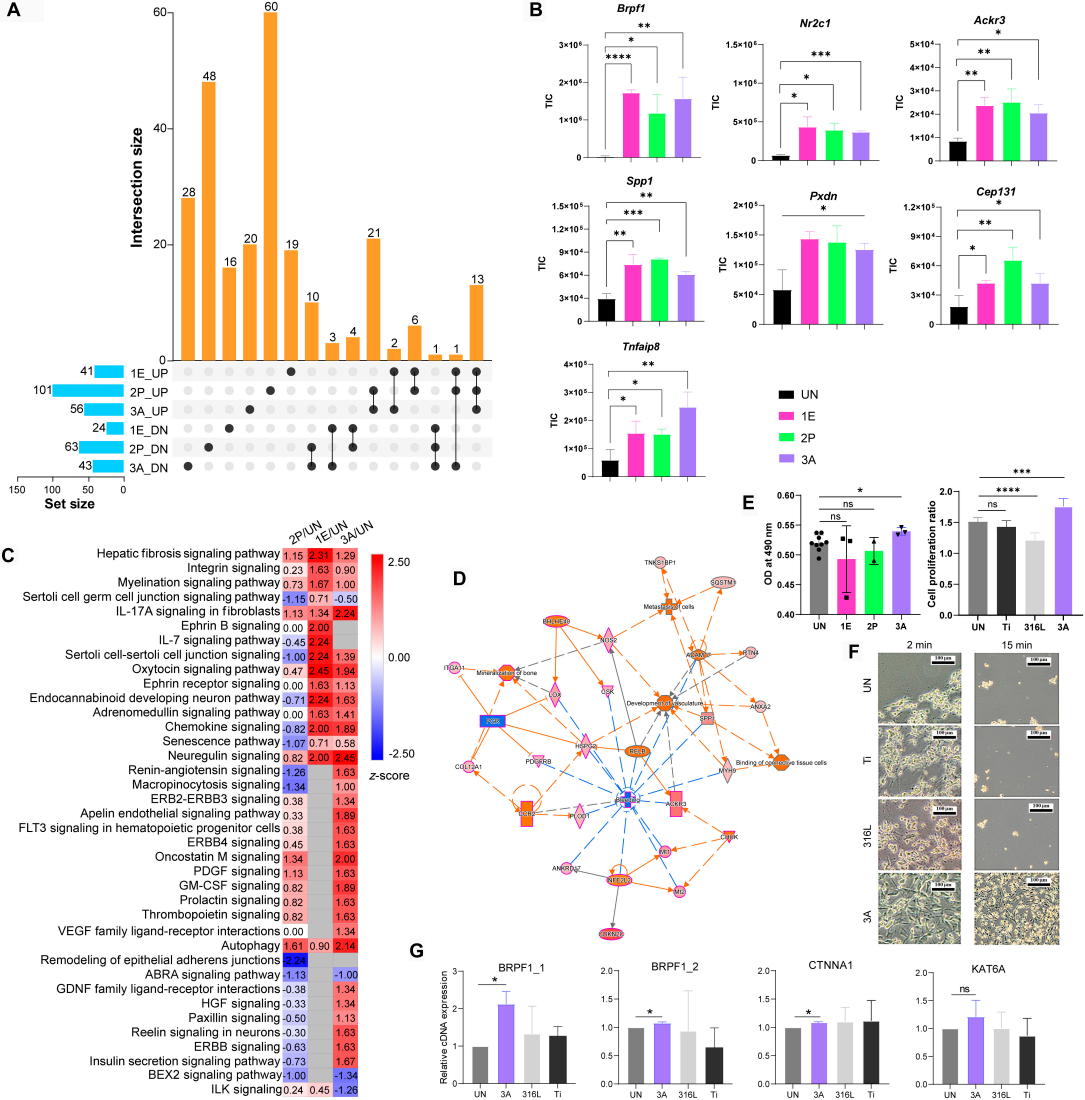
Label-free quantitative proteomic analysis of MC3T3-E1 cells cultured with the presence of Mg alloy specimen after 24 h. (A) Upset plot of up-regulated (including presence only in the alloy groups) or down-regulated (including absence in the alloy groups) DEPs (fold change > 2 and *P* < 0.05) in 3 groups, including 1E, 2P, and 3A, relative to the cell culture without any Mg alloys. The number beside each bar indicates the number of up-regulated or down-regulated DEPs in each group. The black dots and lines represent specific DEPs unique to each alloy group, while the vertical bar chart shows the number of common DEPs shared between alloy groups. (B) The contents (in TIC) of 7 DEPs identified in all groups and up-regulated in the alloy groups and the control (*n* = 3). (C) The canonical pathways associated with “cellular growth, proliferation and development”, “growth factor signaling”, and “organismal growth and development”, differentially regulated by the alloys with respect to the control, suggested by IPA. To discern ‌substantial differences among the groups, a comparative analysis was conducted using a threshold of −log(*P* value) > 1.3. (D) The regulatory network significantly presents only in the 3A group, identified by IPA. Cellular events/canonical pathways/regulators that were activated are indicated by orange, while others that were suppressed are indicated by blue. (E) MTT assay of MC3T3-E1 cells in different alloy groups (left) and in the 3A group in comparison with the Ti and 316L groups (right). (F) Microscopic images of MC3T3-E1 cells in the Ti, 316L, and 3A groups trypsinized for 2 min (left column) and 15 min (right column), respectively, after incubation for 24 h. (G) Comparison of mRNA levels of *Brpf1*, *Ctnna1*, and *Kat6a* by real-time PCR. Ordinary one-way ANOVA with Tukey’s post-hoc test was used in the statistical analysis of (E) and (G). ns, not significant; *P < 0.05; **P < 0.01; ***P < 0.001; ****P < 0.0001 (see Table S3 for detailed proteomic analysis results and Table S2 for statistical results shown in E and G).

In our previous study [3], we revealed that 3A activated fewer inflammation-associated pathways compared to 1E and 2P in a mouse model. Additionally, 3A induced signaling for cell organization and development, suggesting potential benefits for the recovery of injured tissues. Here, we investigate the effects of these materials on mouse osteoblast precursor MC3T3-E1 cells to assess potential impacts on bone development and growth. Proteomic analysis revealed that more differentially expressed proteins (DEPs) were present in 2P, followed by 3A (Fig. 3A and Table S3). Seven up-regulated DEPs were identified across all groups, including BRPF1, NR2C1, ACKR3, SPP1, PXDN, CEP131, and TNFAIP8 (Fig. 3B). Notably, BRPF1 (bromodomain and PHD finger containing 1) showed noticeable up-regulation with 70-, 48-, and 64-fold changes in the 1E, 2P, and 3A groups, respectively. Regarding canonical pathways associated with “cellular growth, proliferation and development”, “growth factor signaling”, and “organismal growth and development”, the highest number of these pathways was activated in the 3A group compared to the untreated group, while less than 50% of these pathways were activated in the 1E group (Fig. 3C). Consequently, “binding of connective tissue cells”, “development of vasculature”, and “mineralization of bone” were enhanced in 3A, while “bleeding” was comparatively inhibited (Fig. 3D).

The proliferation of MC3T3-E1 cells was substantially enhanced, by approximately 16%, in the 3A condition relative to the untreated group, whereas no obvious changes were observed for 1E and 2P, or for the medical Ti or 316L stainless steel groups (Fig. 3E). Notably, 316L markedly inhibited growth of MC3T3-E1 cells. After 24 h culture, a 2-min trypsin digestion was not able to detach MC3T3-E1 cells from the plates in the 3A group, while a suspension of cells was observed for other groups (Fig. 3F). After 15 min of digestion, many cells remained attached to the 3A culture plates, whereas no attachment was observed for the other groups. At the mRNA level, the expression of the 2 mouse Brpf1 isomers was markedly up-regulated in 3A, as was expression of Ctnna1 (Fig. 3G).

### Characterization of coated Mg alloys

**Fig. 4.**
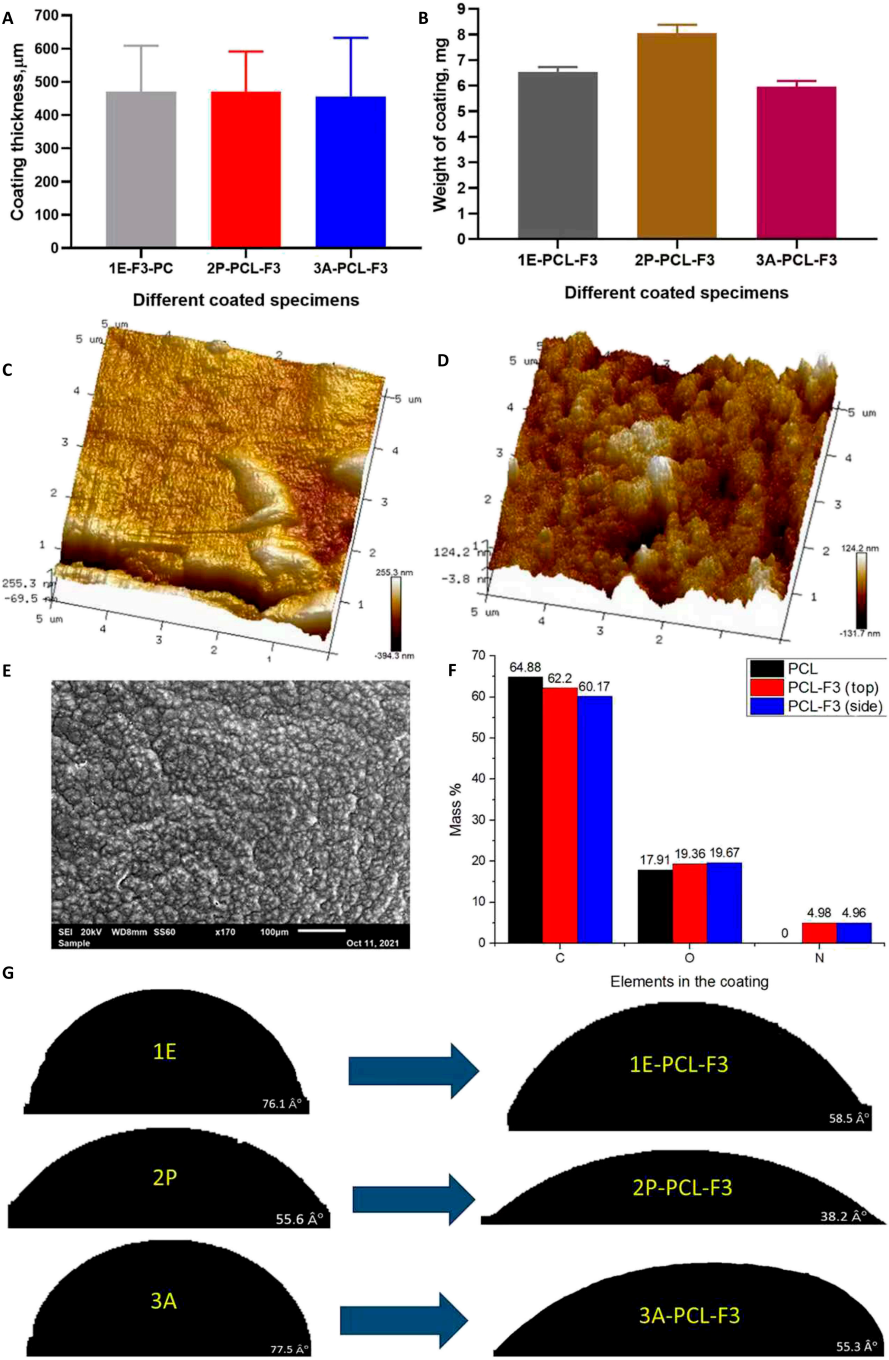
Characterization of coated Mg alloys. (A) Coating thickness of different groups. (B) Weight of different groups. Surface morphology of coated metal (3A-PCL-F3) before (C) and after coating (D). (E) SEM image of PCL-F3 coating. (F) EDX/S analysis of coating elements. (G) Water contact angles of different groups before and after coating.

The characteristics of Mg alloy specimens with the F3 containing PCL coating were studied. All 3 coated metal sample groups had similar coating thickness averaging around 450 μm as shown in Fig. 4A. However, the weights of the coatings differed among the 3 sample groups (Fig. 4B). The 2P-PCL-F3 condition exhibited the highest coating weight with a value of 8.04 mg; 1E-PCL-F3 was next with a weight of about 6.53 mg and 3A-PCL-F3 had the lowest weight of 5.96 mg. The coatings changed the surface morphologies of the samples substantially. Before coating, 3A had regular surface asperities and lower roughness as shown in Fig. 4C. After coating, the surface of 3A in Fig. 4D was markedly rougher, with *R*_a_ and *R*_q_ values increased from 22.8 to 42.4 nm and 31.1 to 66.7 nm, respectively. SEM analysis in Fig. 4E showed that the surface of the PCL-F3 coating developed in this work is as smooth as the PU-F3-coated surfaces in the prior study [2], but its distribution is more uneven and exhibits a regular granular structure on the metal surface. Further, the EDX/S analyses in Fig. 4F show elemental distributions in 3A-PCL-F3. The normal and transverse surfaces of coated samples had almost the same concentrations of nitrogen (4.98% and 4.96%, respectively), oxygen (19.36% and 19.67%, respectively), and carbon (62.2% and 60.17%, respectively). Therefore, it can be deduced that peptide distributions within the coatings were homogeneous.

The results from FTIR analysis of the coated sample surfaces are shown in Fig. S1. At wavelengths around 1,100 cm^−1^, the PCL signal is around 52%T, while for PCL-F3, it is more than 62%T. At a wavelength of 1,150 cm^−1^, the difference between the PCL and PCL-F3 signals becomes more noticeable at around 28%T for PCL and 45%T for PCL-F3. At higher wavelengths (1,750 to 3,300 cm^−1^), differences in the signals from PCL and PCL-F3 become less noticeable.

Assessments of the water contact angles before and after application of the coatings were undertaken to assess hygroscopic properties. They revealed that 2P had the lowest water contact angle of around 55.6° before coating, while after application of the PCL-F3 coating, this was reduced to 38.2°, indicative of the highest hydrophilicity compared with the other alloy samples. Meanwhile 1E and 3A both had similar water contact angles before application of the coating of 76.1° and 77.5°, respectively, which were reduced with the coating to 58.5° and 55.3°, respectively. The results show that the coatings substantially enhance hydrophilicity of the Mg alloy surfaces (Fig. 4G).

### Coated Mg exhibited prolonged bactericidal effects, while in the acute phase, the immune response was activated in all samples, except 3A

**Fig. 5.**
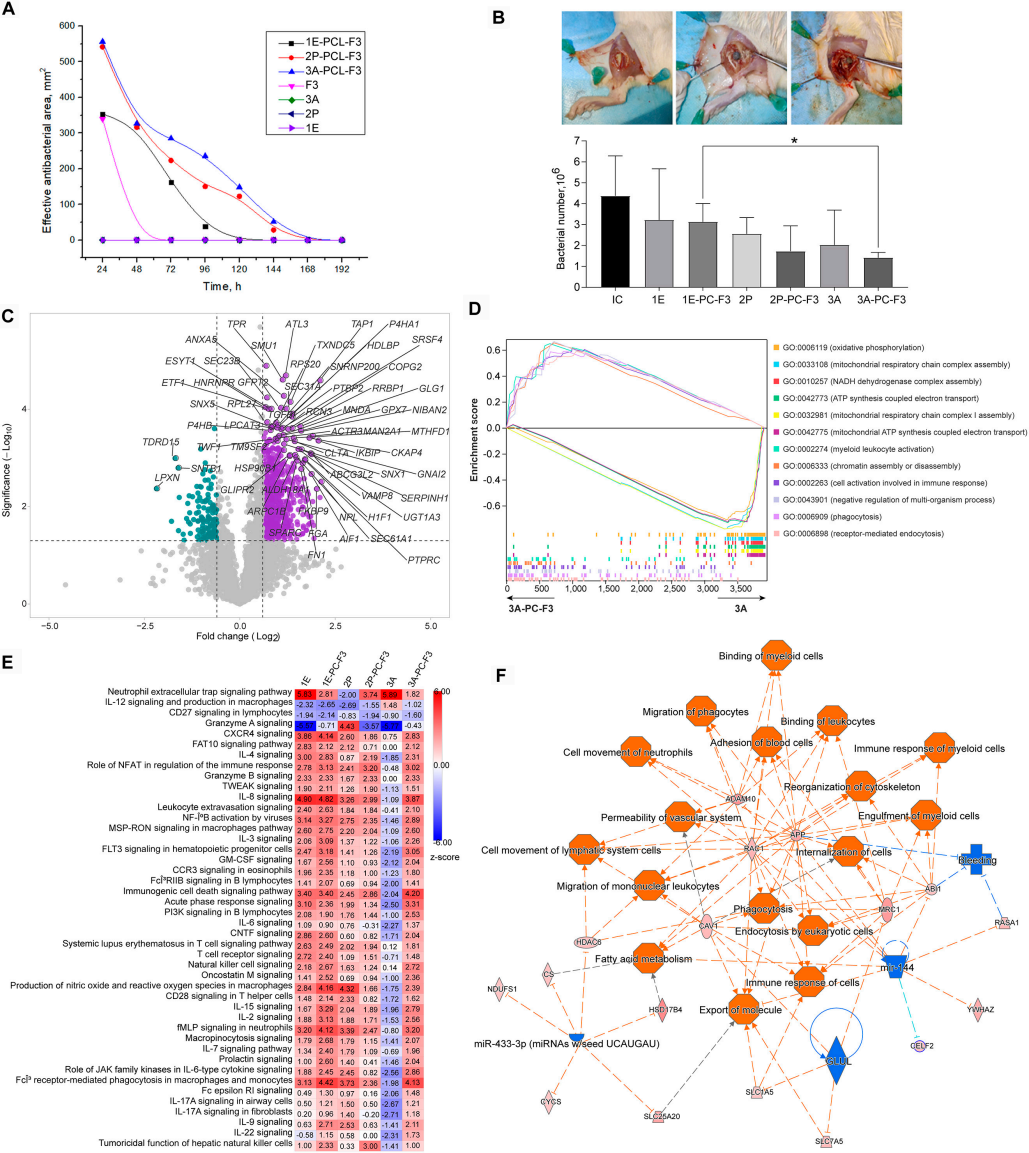
Anti-MRSA properties of Mg alloy implants in vitro and comparative analysis of their implantation effects in vivo at the acute phase of 3 days. (A) Comparison of anti-MRSA activity of different uncoated and coated alloy specimen, with respect to F3 only. (B) Morphology of implantation sites of the control (top left), 3A (top middle), and 3A-PCL-F3 (top right) (see Fig. S3 for the images of other groups), and the concentrations of MRSA in tissues collected from the implantation sites (bottom). (C) Volcano plot comparing the protein contents of 3A-PCL-F3 relative to 3A. Purple dots represent up-regulated DEPs, and green dots represent down-regulated DEPs (fold change > 1.5 or < 0.66, and *P* value < 0.05). (D) Comparison of the top 6 enriched biological processes in 3A-PCL-F3 and 3A by GSEA. (E) IPA compares the activation of immune response relevant pathways in the implant groups with respect to the IC. The black dot indicates insignificance. To discern ‌substantial differences among the groups, a comparative analysis was conducted using a threshold of −log(*P* value) > 1.3. (F) The regulatory network significantly presented only in 3A-PCL-F3, identified by IPA. Cellular events/canonical pathways/regulators that were activated are indicated in orange, while others that were suppressed are indicated in blue (see Table S4 for detailed proteomic analysis results).

The ability to counter MRSA-related infection was assessed in vitro for both uncoated and coated Mg alloys, and the results were compared to the peptide, F3, which displayed bactericidal activity for up to 72 h. The uncoated alloys exhibited no discernible impacts on MRSA infection, while all 3 coated Mg alloys demonstrated prolonged antibacterial resistance (Fig. 5A). Specifically, 1E-PCL-F3 exhibited bactericidal effects lasting up to 120 h, whereas 2P-PCL-F3 and 3A-PCL-F3 inhibited MRSA up to 168 h, with the latter showing a slower decline in efficacy over time. Figure 5B shows the implantation sites, with 3A and 3A-PCL-F3 causing no obvious inflammation during the acute stages. The numbers of MRSA isolated from tissues of similar weight collected during the acute phase were least in 3A-PCL-F3. Noticeable differences were observed between 3A-PCL-F3 and 1E-PCL-F3, consistent with the in vitro assay findings. Results have confirmed that F3 does not induce resistance to logarithmic *Pseudomonas aeruginosa* and MRSA after 3 months of culture; hence, implantation of the PCL-F3-coated Mg samples is not expected to induce resistance to MRSA in the longer term.

Compared to the noninfected control (NIC), 2P showed the highest number of up-regulated DEPs, followed by 3A-PCL-F3 and 1E-PCL-F3, with a similar trend observed in comparison with the infected control (IC) (Fig. S2A, Fig. 5B, and Table S4). 2P and 3A-PCL-F3 shared higher numbers of mutual up-regulated DEPs compared to the NIC and IC. In 3A-PCL-F3, a total of 756 proteins were ‌markedly‌ up-regulated, and 138 proteins were down-regulated compared to 3A. Among them were many proteins associated with cell homeostasis and tissue growth including MTHFD1L, SPARC, ATL3, TGFBI, and SEC61A1 (Fig. 5C). Trend analyses identified 4 profiles with significance (*P* value < 0.05). Profile 17 exhibited 646 proteins with similar quantitative features in the 1E, 1E-PCL-F3, 2P, and 3A-PCL-F3 (Fig. S2C). This profile exhibited the enrichment of immune response-relevant biological processes with the lowest FDR values, such as “response to stress”, “defense response”, and “immune system response” (Fig. S2D). Several biological processes related to mitochondrial function were among the top GO terms enriched in 3A, while processes possibly supporting an antibacterial environment, such as “phagocytosis” and “receptor-mediated endocytosis”, were highly enriched in 3A-PCL-F3 (Fig. 5D). The noticeable up-regulation of AIF1, TXNDC5, MYO1G, ITGB2, STXBP2, VAMP8, ITGAM, and ANXA1 supported the activation of “phagocytosis” (Fig. S2E) and “cell activation involved in immune response” (Fig. S2F), respectively.

Assessment of activation of the signaling response pathways in all implant groups relative to the IC revealed a distinctive feature in 3A, which showed obvious suppression of pathways associated with T cell function. These included the signaling of IL-8, IL-15, IL-2, IL-4, CD28, and CCR3 (Fig. 5E). Conversely, most of these showed increased activity in the other implant groups. Unique activation of the “IL-12 signaling and production in macrophages” pathway was observed in 3A. Additionally, the up-regulated DEPs supported noticeable activation of “phagocytosis” specifically in 3A-PCL-F3 compared to the IC (Fig. 5F). Furthermore, several other pathways potentially associated with bactericidal activity, such as “immune response of myeloid cells”, “engulfment of myeloid cells”, and “migration of phagocytes”, were also activated in 3A-PCL-F3, while “bleeding” was ‌markedly‌ inhibited, suggesting potential favorable impacts on wound healing.

### Uncoated and coated 3A showing better biocompatibility

**Fig. 6.**
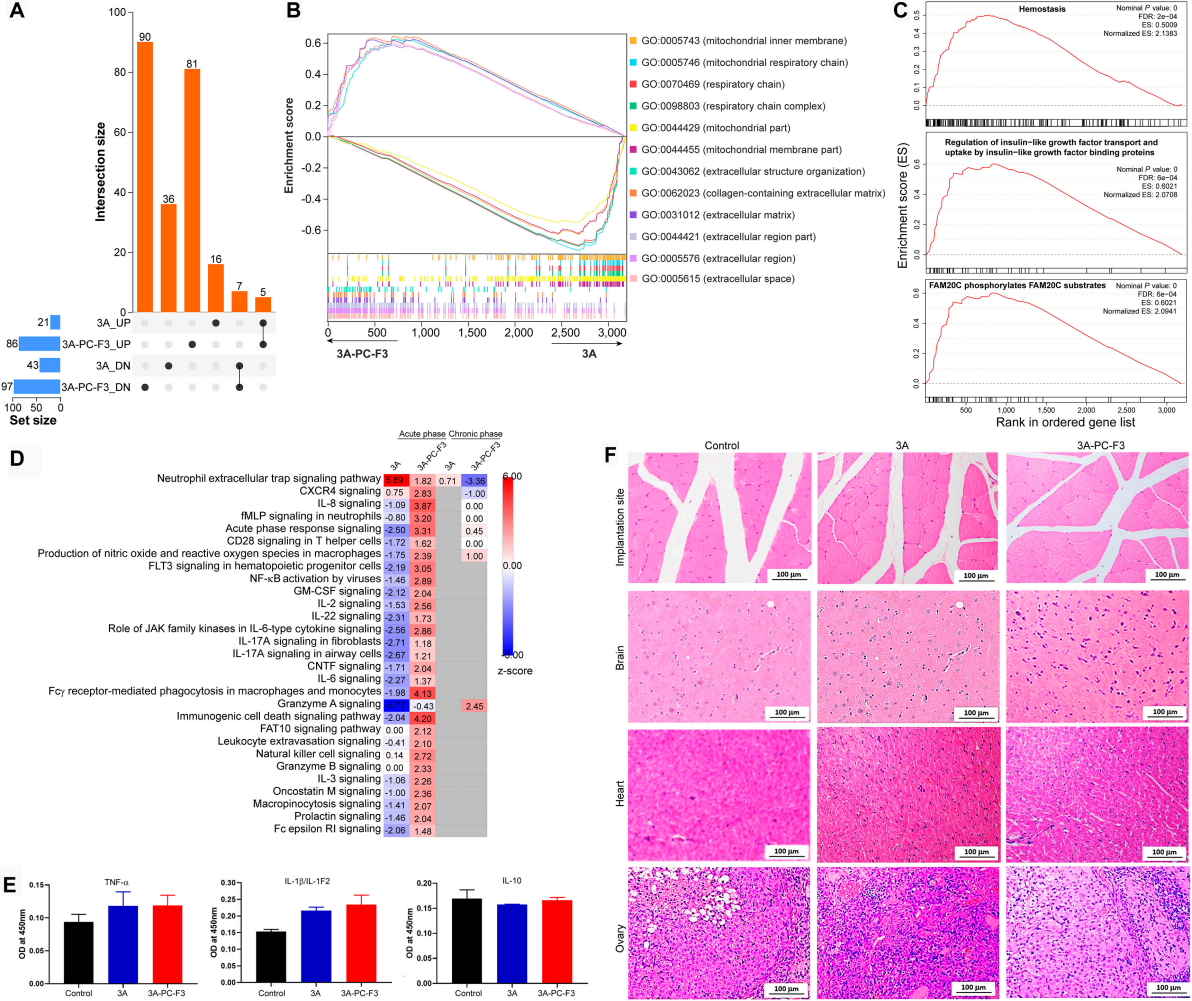
Comparative proteomic analysis of tissues collected from 3A and 3A-PCL-F3 at 3-month post-implantation and HE-stained histological analysis. (A) Upset plot of up-regulated or down-regulated DEPs (fold change > 1.5 or < 0.67, and *P* < 0.05) in 3A and 3A-PCL-F3 relative to the NIC. The number beside each bar indicates the number of DEPs in each group. The black dots and lines represent specific DEPs unique to each implant group, while the vertical bar chart shows the number of common shared DEPs between groups. (B) GSEA depicting the top 6 enriched cellular components by DEPs in 3A-PCL-F3 and 3A, respectively. (C) Top 3 most noticeable biological processes in 3A-PCL-F3 compared to 3A. (D) IPA of immune response relevant pathway regulation in implant groups at acute (3 days) and chronic (3 months) phases relative to the control. To discern significant differences among the groups, a comparative analysis was conducted using a threshold of −log(*P* value) > 1.3. (E) ELISA analysis of TNF-α, IL-1β/IL-1F2, and IL-10 in the control, 3A, and 3A-PCL-F3. (F) HE-stained tissues collected from the implantation sites, brain, heart, and ovary of rats in the control, 3A, and 3A-PCL-F3 (see Table S5 for detailed proteomic analysis results).

There were more ‌markedly‌ up- or down-regulated DEPs in 3A-PCL-F3 compared to 3A with respect to the NIC (Fig. 6A and Table S5). However, the overlap of DEPs between these 2 groups was limited. Notably, the biological processes enriched in 3A-PCL-F3 encompassed a multitude of metabolic activities associated with mitochondrial function. These included processes such as the “tricarboxylic acid cycle”, “mitochondrial electron transport, cytochrome c to oxygen”, “proton transmembrane transport”, and “mitochondrial ATP synthesis coupled electron transport” (Fig. S4A). In contrast, 3A exhibited enrichment in developmental processes, such as diaphragm, seminal vesical epithelium, and seminal vesicle development (Fig. S4B). Many DEPs up-regulated in 3A-PCL-F3 play pivotal roles in cell growth and tissue repair, such as FBLN2, IGHM, CLEC3B, SUN1, and COL5A2 (Fig. S4C). Furthermore, up-regulated DEPs exhibited extensive interactions. Notably, proteins like STAT3, TUBB2B, ARF5, SOD1, and CANX displayed the highest connectivity. These proteins are mainly involved in regulating immune responses and cellular trafficking (Fig. S4D).

There were important differences in the cellular components associated with the proteins in the 3A and 3A-PCL-F3. Proteins identified in the former were primarily linked to mitochondrial components, whereas the latter displayed enrichment of proteins in the extracellular region (Fig. 6B). The top 6 most noticeably enriched biological processes in 3A-PCL-F3 were all closely tied to cell growth and development. These processes encompassed “regulation of insulin-like growth factor transport”, “FAM20C phosphorylates FAM20C substrates”, and “hemostasis” (Fig. 6C and Fig. S4E). Comparing immune response-relevant pathways at the chronic phase with those at the acute phase, a notable reduction in the degree of activation was observed in both 3A and 3A-PCL-F3, with respect to the NIC (Fig. 6D). Only one pathway with a *z*-score > 2, granzyme A signaling, was activated in 3A-PCL-F3, while the “neutrophil extracellular trap signaling pathway” was ‌markedly‌ and exceptionally suppressed. The extent of activation was substantially lower compared to the response at the acute phase when the implantation of 3A-PCL-F3 led to ‌noticeable activation of immune responses. Blood samples from the 3A and 3A-PCL-F3 were used to evaluate IL-10, TNFα, and IL-1β levels, which appeared similar and indicated no systemic inflammation in the various implant groups compared to the control (Fig. 6E). Notably, no obvious pathological changes were detected in the tissues from the implantation sites, brain, heart, and ovaries, and tissues displayed normal morphological features (Fig. 6F).

### Muscle cell proliferation was induced in 3A in the acute phase of bacterial infection, while metabolism of cholesterol derivatives was enhanced during the chronic phase

**Fig. 7.**
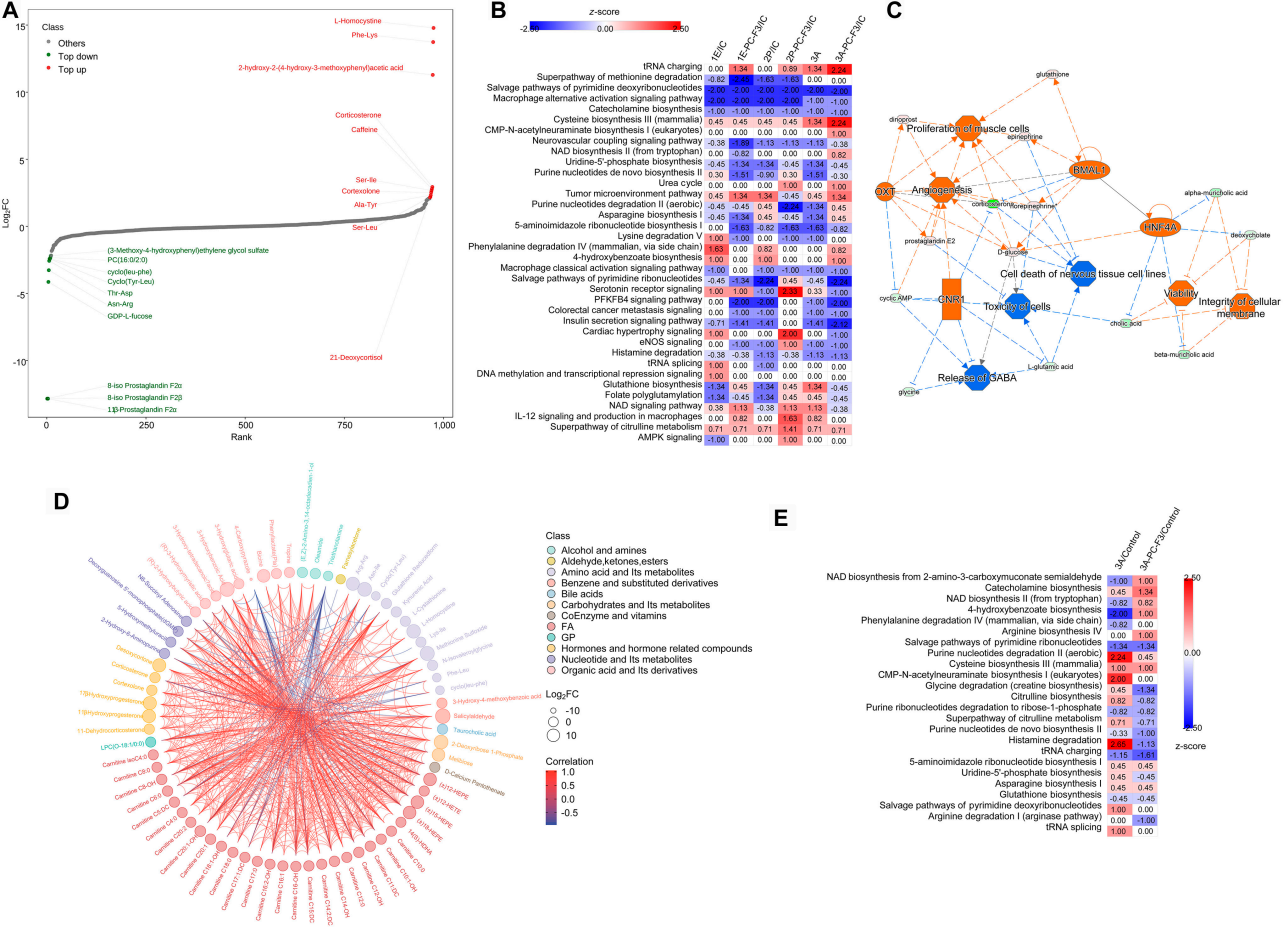
Metabolomic analysis of tissues collected from the implantation sites of coated and uncoated Mg alloys in the acute and chronic phases with the introduction of MRSA bacteria. (A) Top 20 DEMs (differentially expressed metabolites) identified in 3A-PCL-F3 compared to 3A. (B) IPA comparing regulation of metabolic pathways in the implant groups relative to the IC at the acute phase. To discern significant differences among the groups, a comparative analysis was conducted using a threshold of −log(*P* value) > 1.3. (C) The regulatory network ‌markedly‌ presents only in 3A-PCL-F3, identified by IPA. Cellular events/canonical pathways/regulators that were activated are indicated in orange, while others that were suppressed are indicated in blue. (D) Correlations among the DEMs of different classes in 3A-PCL-F3. (E) IPA comparing regulation of metabolic pathways in 3A and 3A-PCL-F3 with respect to the NIC in the chronic phase (see Table S6 for detailed metabolomic analysis results in the acute and chronic phases, respectively).

Extracted metabolites from tissues at the implant sites were subjected to LC-MS/MS analysis assessed with respect to the IC in the acute phase. Metabolites such as L-homocystine, Phe-Lys, and 2-hydroxy-2-(4-hydroxy-3-methoxyphenyl) acetic acid showed remarkable up-regulation, while there was distinct down-regulation of several prostaglandins associated with oxidative stress and inflammation was in 3A (Fig. 7A). ‌Noticeable regulation of multiple metabolic pathways was observed in the Mg alloy sample groups compared to the IC, with many more of them being suppressed than activated (Fig. 7B). The pathway that saw the most deactivation across all implants was the “salvage pathways of pyrimidine deoxyribonucleotides”. Likewise, the biosynthesis of uridine-5′-phosphate, catecholamine, citrulline, and “histamine degradation”—essential pathways for DNA and RNA synthesis, neurotransmission, and the urea cycle—was also inhibited. Of note, the 3A-PCL-F3 group exhibited heightened activation of “tRNA charging”, “cysteine biosynthesis”, “NAD biosynthesis II (from tryptophan)”, and “CMP-N-acetylneuraminate biosynthesis I”, in comparison to the other implant groups. In particular, the regulatory network supported the activation of “proliferation of muscle cells” and “angiogenesis”, while the suppression of “toxicity of cells” and “nervous tissue cell death” was only ‌markedly‌ identified in the 3A-PCL-F3 group (Fig. 7C). Examining KEGG pathways, it was evident that “steroid hormone biosynthesis”, “cortisol synthesis and secretion”, and the aberrant production of glucocorticoids (“Cushing syndrome”) were enriched in 3A-PCL-F3 (Fig. S5A). Four regulatory networks of significance were identified in 3A-PCL-F3 compared to the 3A group. Among these, the abnormal choline metabolism pathway was extensively regulated by 6 enzymes and 2 modules, interconnected by 2 differentially expressed metabolites (DEMs), namely, cytidylic acid and citicoline (Fig. S5B). The other 3 enriched networks involved conversion between [NAD(P)+] and NADPH, as well as C21-steroid hormone biosynthesis, utilizing the energy produced during the conversion of NADH to NAD+.

We observed a more than 10-fold up-regulation of 15 metabolites in 3A-PCL-F3 compared to 3A. Noteworthy among these were salicylaldehyde, 3-hydroxybenzoic acid, and methionine sulfoxide (Fig. S5C). The up-regulated DEMs from 12 classes displayed strong positive correlations, with many belonging to fatty acids and amino acid metabolites (Fig. 7D). Conversely, down-regulated DEMs exhibited significantly lower correlations with others and encompassed compounds like 4-carboxypyrazole, triethanolamine, cyclo (Tyr-Leu), and farnesylacetone. The biosynthesis of NAD, catecholamine, and arginine were activated in 3A-PCL-F3 compared to the control, surpassing regulation observed for 3A (Fig. 7E). Important canonical pathways activated in 3A included “histamine degradation”, “purine nucleotides degradation II”, and “CMP-N-acetylneuraminate biosynthesis I”. Interestingly, the sole immune response relevant signaling pathway activated in both 3A and 3A-PCL-F3 derived from the DEMs was the “macrophage alternative activation signaling pathway” (Table S6). An enrichment of KEGG pathways linked to cholesterol metabolism was identified in 3A-PCL-F3 relative to 3A such as “steroid hormone biosynthesis”, “cortisol synthesis”, and “secretion and bile secretion” (Fig. S5D), which accorded with activation of NAD biosynthesis. Consequently, this led to the identification of 2 closely regulated networks (Fig. S5E). Six DEMs modulated 10 enriched KEGG pathways relevant to steroid hormones and lipolysis, while 8 DEMs were associated with amino acid and cholesterol metabolisms.

## Discussion

Previously, we confirmed that 3A- and PCL-F3-coated 3A showed obvious improved corrosion resistance compared to pure Mg (2M) and cold extruded AZ31 (2E) [3]. Also, we already checked and confirmed the binding force between the coating and the AZ31 base in the previous work [2]. In this study, we characterized a series of Mg biomaterials (pure Mg, cold rolled AZ31, and fully annealed AZ31), both uncoated and coated with PCL incorporating caerin F3 HDP. We investigated their in vitro and in vivo (in SD rats) antibacterial performances, as well as effects on tissues at the implantation sites, using proteomic and metabolomic analysis. Notably, 3A-PCL-F3 exhibited remarkable in vitro antibacterial effects, which continued up to 144 h. This was accompanied by activation of diverse inflammatory responses, especially phagocytosis activated in vivo during the acute phase. Furthermore, 3A-PCL-F3 also demonstrated superior biocompatibility, with limited immunoregulatory effects and enhanced NAD biosynthesis at 3 months post-implantation. Importantly, 3A promoted mouse osteoblast precursor cell MC3T3-E1 proliferation through up-regulating expression of Brpf, a gene with the ability to promote bone formation.

### 3A effectively promoted proliferation of murine osteoblastic cells

In previous research, quantitative proteomic analysis revealed that 3A activated pathways linked to wound healing and tissue development [3]. Building on this, the impacts of Mg alloy biomaterials on mouse osteoblast precursor MC3T3-E1 cells were assessed. Notably, BRPF1 was obviously up-regulated in 3A, in both protein and mRNA levels. BRPF1 is a crucial component of multiprotein complexes involved in histone acetylation, playing a pivotal role in gene expression regulation through chromatin remodeling [30]. It has been found that BRPF1 is important for murine neural stem cell development [31,32], and its deletion led to bone marrow failure [33]. Additionally, the inhibition of pan BRPF bromodomain suppresses transcriptional programs required for osteoclastogenesis, in both mice and humans [34]. Thus, the up-regulation of BRPF1 suggests that enhanced osteoclast differentiation was induced by 3A, which potentially supports bone tissue repair and remodeling in vivo.

Up-regulation of BRPF1 in 3A is reflected in the pronounced activation of signaling pathways related to “binding of connective tissue cells”, “development of vasculature”, and “mineralization of bone”, alongside the deactivation of “bleeding”. Compared to the other Mg alloys and commonly used metallic biomaterials (Ti and 316L), 3A markedly promoted MC3T3-E1 cell growth, as supported by the MTT assay, and also promoted their adhesion properties, indicating potential alterations in expression of molecules related to adhesion and extracellular matrix (ECM) composition. The resistance to trypsin in MC3T3-E1 cells, capable of differentiating into osteoblasts, suggests that osteogenic differentiation was induced by 3A. In addition, osteoblasts interact closely with the ECM and can form multicellular structures [35], which may also hinder detachment. Moreover, 2 proteins, PDLIM4 and RINT1, were present with relatively high abundance in 3A compared to 1E and 2P. PDLIM4 was suggested to play roles in the organization of protein complexes and the regulation of cytoskeletal dynamics [36], while RINT1 is associated with DNA repair and genome maintenance [37]. Thus, 3A exhibits strong potential to promote osteocyte growth in vivo by orchestrating collective regulation of multiple proteins that support cell proliferation and tissue development.

### 3A did not stimulate inflammatory responses in the acute and chronic phases and coating with PCL-F3 did not obviously alter biocompatibility

During the acute phase, after the introduction of MRSA with implantation, 3A demonstrated unique suppression of signaling pathways associated with inflammatory responses. Proteomics revealed that only 2 immune response pathways were obviously activated, namely, the “neutrophil extracellular trap signaling pathway” and “IL-12 signaling and production in macrophages”. This suggests distinctive immunomodulatory effects induced by 3A, leading to enhanced anti-inflammatory responses associated with immune tolerance and reduced tissue damage or foreign body reaction [24]. Distinctively, 3A induces fewer inflammatory responses after implantation compared with the other investigated alloys, 1E and 2P. 2P, being pure Mg, exhibits higher chemical reactivity, leading to an increased inflammatory response. However, despite 1E and 3A exhibiting similar chemical compositions and rough surface, they induce substantially different inflammatory responses. Differences in surface texture may have a role with EBSD analysis revealing that 3A has a higher proportion of first-order pyramidal (10–11) {10–1–2} texture than 1E [3], which may induce different surface physiochemical properties including differences in surface energy, corrosion, surface interactions, and bonding [2]. Other mechanical properties, such as strength and plasticity, may also impact host responses, which warrants further investigation.

The PCL-F3 coating imparted 3A with better antibacterial performance, evident in enhanced phagocytosis and other bactericidal process pathways. Of particular interest in 3A-PCL-F3, the protein, TAP1, showed the most ‌noticeable up-regulation. TAP1 plays a pivotal role in the immune system by transporting cytosolic peptides into the endoplasmic reticulum (ER), enabling MHC class I molecules to present these peptides on the cell surface for recognition by cytotoxic T cells [38]. It may work in conjunction with another highly up-regulated protein, SEC61A1, responsible for protein translocation into the ER lumen [39]. Similarly, CKAP4, localized to the ER membrane, aids in protein trafficking [40], indicating its role in phagocytosis by facilitating the uptake of pathogens. At the metabolite level, tRNA charging and cysteine biosynthesis were greatly enhanced in 3A-PCL-F3 compared to the other materials, indicating an elevation in translation.

In the chronic phase after implantation, immune response regulation pathways detected in 3A resembled that of tissue recovery without an implant, which showcases its outstanding biocompatibility. Signaling pathways in 3A-PCL-F3 were enriched in the extracellular region, whereas in 3A, enrichment was observed in mitochondria. The regulation of immune response signaling in both 3A and 3A-PCL-F3 was minimal, although gene for granzyme A (GZMA) signaling remained activated in the latter group. This suggests that the degradation of PCL and F3 potentially contributed to cell–cell communication and adherence, whereas Mg^2+^ predominantly influenced energy metabolism. Various types of PCL have been employed as scaffold materials to facilitate growth of cells and tissues and aid tissue regeneration [41]. Additionally, degradation of PCL was found to provide temporary support for tissue growth [42]. Therefore, the gradual degradation of PCL could potentially offer prolonged mechanical support in bone regeneration during the chronic phase of recovery. Of significance, most immune response pathways activated during the acute phase in 3A-PCL-F3 diminished notably at the chronic phase. Considering that GZMA is involved in various immune responses, particularly in eliminating infected or abnormal cells, the activation of GZMA signaling could potentially support ECM remodeling and tissue development [43]. Furthermore, at the metabolite level, the enhanced biosynthesis of NAD in 3A-PCL-F3 indicated increased energy production, which was reflected in elevated levels of various carnitines. This implies potential enhancement of cell and tissue development, indicating comprehensive biocompatibility for both 3A and 3A-PCL-F3. Collectively, it can be postulated that as the PCL-F3 coating degrades at the acute phase, it activates inflammatory signaling pathways and controls bacterial infection, while in the chronic phase, the physical characteristics of 3A suppress foreign body reactions.

### 3A-PCL-F3 demonstrated better in vitro and in vivo antibacterial performance

Various antibacterial mechanisms have been observed in coatings employing HDPs, including the destruction of the bacterial membrane, blocking DNA replication, inhibiting adenosine triphosphate synthase, impeding cell respiration, and disrupting protein synthesis. While many antibacterial coatings have demonstrated effective antibacterial properties against *S. aureus* [43] or *Escherichia coli* [44], or both [45], there is a scarcity of research on the antibacterial abilities of coated magnesium alloys against stubborn drug-resistant bacteria. Previously, our study confirmed that 3 Mg alloys (1E, 2P, and 3A) did not show any antibacterial behaviors in vitro and in vivo, but compared to 1E and 2P, 3A showed improved corrosion resistance both in vitro and in vivo [3]. In this study, PCL-F3 coating showed pronounced and enduring inhibitory effects on MRSA by targeting membrane integrity both in vitro and in vivo. For in vitro tests, the antibacterial effects persisted for approximately 6 days (144 h). Furthermore, after 73 h of implantation, tissues collected from the implantation sites still inhibited MRSA.

**Fig. 8.**
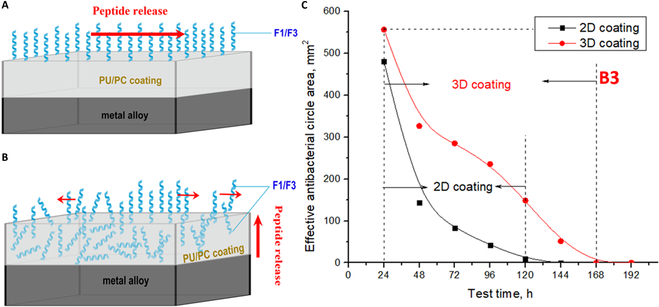
Schematic showing mode of action of 2D and 3D antibacterial polymer coatings. (A) 2D antibacterial coating. (B) 3D antibacterial coating. (C) Comparison of 2D [2] and 3D antibacterial coatings.

The sustained release of peptides from the coating is pivotal for the antibacterial efficacy. In our previous study [2], click chemistry immobilization of the F3 peptide on the surface of Mg alloys provided a 2D PU coating on the metal alloy surface (Fig. 8A). In the current work, dip coatings of the PCL were employed as the polymeric “scaffold” to create a 3D coating with F3 entrained within. The 2D coating covalently immobilizes one terminus of the peptide to the alloy’s surface, while the 3D coating allows the peptides to reside within the coating layer on the surface of the alloy (Fig. 8B). The in vitro antibacterial assessments confirmed enhanced antibacterial effects from the 3D coating, which displayed a larger bacteria-free area and longer duration of action compared to the 2D coatings (Fig. 8C) [2]. Theoretically, the 3D coating enables greater loading of the peptides. Moreover, for the 2D coating, once the peptides are consumed by either the physiological environment or the bacteria through enzymatic degradation, there is no capacity to supplement areas where peptides have been consumed, potentially reducing the overall efficacy.

The intricate surface structure formed through the combination of PCL and F3 establishes a dense matrix. This matrix interacts with the peptides, restricting mobility and presenting a challenge for them to migrate from regions of high concentration to low concentration. Consequently, this results in a gradual, sustained release of the peptides from the coated surface. In addition, the combination of slow release and strengthened interaction inside the 3D coating facilitates F3 movement along concentration gradients, contributing to stable and continuous antibacterial effects, which endured for almost 7 days. The increased loading of the F3 peptide within the 3D coatings contributes to the sustained action. Further research to understand the mode of action and optimize the coating’s performance is ongoing.

### 3A-PCL-F3 showed better biocompatibility

In addition to antibacterial effects and corrosion resistance, biocompatibility is a critical factor in the performance of the coatings. Recently, Qian et al. [46] used a solution containing Cu^2+^ and phosphate to seal a plasma electrolytic oxidation-coated AZ91D Mg alloy and acquired enhanced corrosion resistance and antibacterial properties in vitro. However, there are potential issues of cytotoxicity and biocompatibility due to the introduction of Cu^2+^ and phosphate when the coated Mg alloy is planted. In 2024, Pesode et al. [47] developed an antibacterial coating on Mg alloy by the micro-arc oxidation surface modification technique; the coated Mg alloys obtained improved corrosion resistance and antibacterial behavior in vitro. The study did not check the in vivo behaviors of the coated Mg alloy, as well as biocompatibility. Reducing and limiting FBRs is an essential goal for implants, and various technologies, such as thermal spraying, chemical conversion, and biomimetic approaches [48], have been employed to obtain protective antibacterial coatings. Common polymer-based antibacterial coatings include calcium phosphate-based coatings [42]. For polymer-based antibacterial coatings, biodegradable polyesters including polylactic acid, polyglycolic acid, polylactic acid-co-glycolic acid, and their copolymers have demonstrated promising results (including antibacterial behaviors and biocompatibilities) when incorporating antibacterial elements onto Mg alloy coatings. PCL has been confirmed as an effective polymer medium for coating to further improve the corrosion resistance and biocompatibility of Mg [49], capable of physically or chemically interacting with F3 in the coating, likely via H-bonding.

As a key measure of biocompatibility, cytocompatibility assesses the impact of coated metals on cell proliferation. Previously, cell studies frequently demonstrated positive effects on cytocompatibility and cell proliferation from antibacterial coatings, particularly for bone-linked cells such as murine osteoblast-like cell line MC3T3-E1 and human osteoblast cell line MG-63 [50]. These studies assess proliferation in conjunction with cell adhesion and osteoblast differentiation. In this study, 3A-PCL-F3 showed substantially enhanced proliferation, osteoblast differentiation, and adhesion of mouse osteoblast precursor MC3T3-E1 cells over other materials. Additionally, tissues collected from the implantation sites, brain, heart, and ovary of rats demonstrated that 3-month post-implantation with 3A-PCL-F3, there was no detectable impact on important organs. Furthermore, the implantation of 3A-PCL-F3 did not induce any obvious immune or inflammatory responses, which have not been reported previously for other antibacterial coatings. The cytokine levels from the sera 3 months post-implantation were similar among the control group, 3A, and 3A-PCL-3A, which further indicates its outstanding biocompatibility.

### Future research directions

The current findings reinforce our previous observations that fully annealed AZ31 Mg alloy (3A) exhibits superior biocompatibility, supports tissue recovery, and contributes to osteocyte regeneration and development. When coated with PCL and the HDP caerin 1.9 (F3), the 3A alloy (3A-PCL-F3) demonstrated greatly enhanced antibacterial efficacy and biocompatibility both in vitro and in vivo. However, the underlying reasons for the distinct anti-inflammatory and regenerative behaviors observed in 3A compared to its nonannealed counterpart remain unclear. The annealing process, including parameters such as heating speed, annealing temperature, holding time, furnace atmosphere, and cooling speed, may substantially affect the resulting microstructure and phase distribution in the alloy. Future investigations will focus on understanding how these thermal treatment parameters influence microstructural features, surface physicochemical properties, and the corresponding in vivo responses.

Interestingly, 3A outperformed cold-extruded AZ31 (1E) in terms of antibacterial effects, despite both alloys sharing similar chemical composition and surface characteristics. This suggests that the nature of interactions, whether physical or chemical, between the metal substrate and the polymeric coating could influence antibacterial performance. Further studies are needed to elucidate the interfacial structures formed between the PCL-F3 coating and Mg alloy substrates and to clarify the specific mechanisms driving the bactericidal activity.

Caerin 1.9 is known to exert its antibacterial effect by forming pores in the membranes. Although it generally exhibits a short half-life of approximately 1.16 h in vivo, encapsulation within PCL coatings appears to extend its functional lifespan to over 3 months [22]. Nevertheless, the kinetics of peptide release in physiological conditions remains inadequately characterized. Future work will focus on quantifying the release kinetics understanding the mechanisms of sustained antibacterial activity within the implant microenvironment. Due to limitations in current facilities, standard CT was employed in this study, which cannot fully resolve the histological features at the bone–implant interface. In upcoming research, advanced tools such as high-resolution micro-CT, x-ray diffraction, and multi-omics approaches (proteomics, metabolomics, genomics) will be employed in the longer-term studies (12 months or longer) to provide deeper insight into the tissue integration and immunological responses. The histopathological evaluation will be conducted in collaboration with clinical pathologists at a partner hospital. We also plan to develop and assess dual-functional coatings that combine antimicrobial peptides with osteoinductive agents to simultaneously prevent infection and promote osseointegration. In parallel, mechanical stability of the coatings under physiological loading and dynamic conditions will also be systematically investigated.

## Conclusion

In summary, this study successfully developed a novel 3D PCL-based coating incorporating the cationic HDP caerin 1.9 (F3) to enhance biocompatibility and antibacterial performance of degradable Mg alloy implants. The PCL-F3-coated Mg alloys exhibited excellent in vitro and in vivo antibacterial efficacy, sustained peptide release, and superior biocompatibility, without inducing adverse effects on major organs in SD rats up to 3 months post-implantation. Among the tested materials, fully annealed AZ31 alloy (3A) exhibited the most favorable outcomes. Proteomics and metabolomics analyses revealed that 3A did not activate chronic inflammation or immune responses but instead promoted cell proliferation and up-regulated pathways involved in tissue regeneration and hemostasis. Unlike pure Mg (2P) and cold extruded AZ31 (1E), 3A-PCL-F3 implants triggered minimal foreign body responses and demonstrated superior integration with host tissues. In vitro studies using MC3T3-E1 pre-osteoblast cells further confirmed the osteogenic potential of 3A, showing enhanced cell proliferation and up-regulation of BRPF1 protein and other key regulators of bone mineralization. Histological and functional assessments confirmed that 3A-PCL-F3 implants maintained hepatic and renal health and activated transient phagocytic immune responses during the acute phase (within 72 h), supporting tissue repair while avoiding long-term immune complications. Overall, the 3A-PCL-F3 composite exhibits a unique combination of long-lasting antibacterial protection, osteogenic stimulation, and excellent biocompatibility, making it a highly promising candidate for the next generation of degradable biomedical implants.

## Ethical Approval

All experimental procedures were approved and conducted in accordance with the guidelines of the Animal Experimentation Ethics Committee (Ethics Approval Number: C202307-5) by the Foshan First People’s Hospital and the University of the Sunshine Coast’s Animal Ethics Committee (Ethics Approval Number: ANE23105).

## Data Availability

All data are available in the main text or the Supplementary Materials.
