# Supplementary material for: Degradable Magnesium Implants with Caerin 1.9-Polycaprolactone Coatings Provide Extended Antibacterial Resistance and Outstanding Biocompatibility
Source: Biomater Res. 2025 Oct 28;29:0257. doi: 10.34133/bmr.0257 (PMC12559799; doi:10.34133/bmr.0257)
Supplement: Supplementary 1 — Figs. S1 to S5 Tables S1 to S6 [file bmr.0257.f1.zip › supplementary materials.docx]

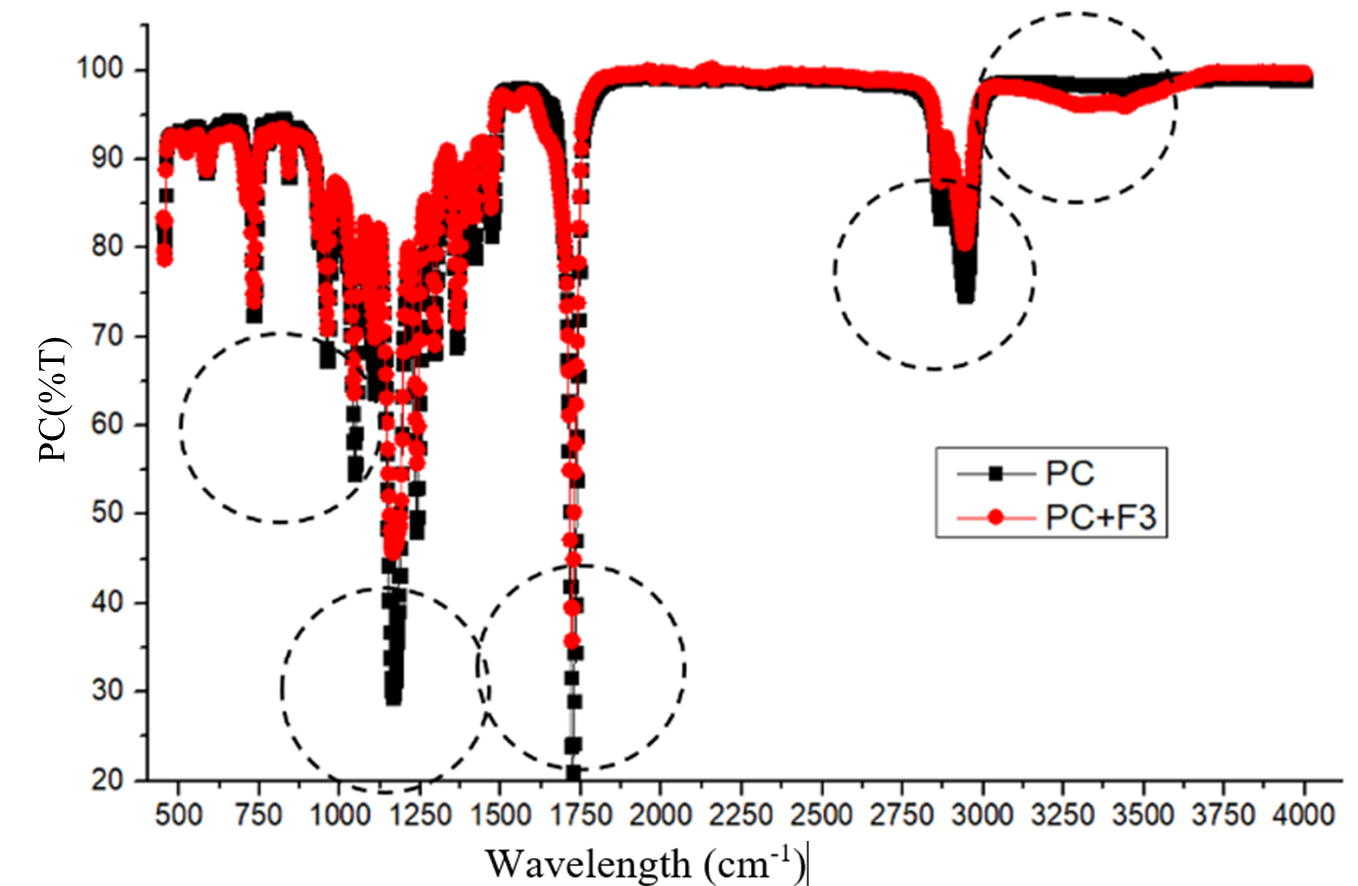


Fig. S1. FTIR analysis of PCL and PCL-F3.


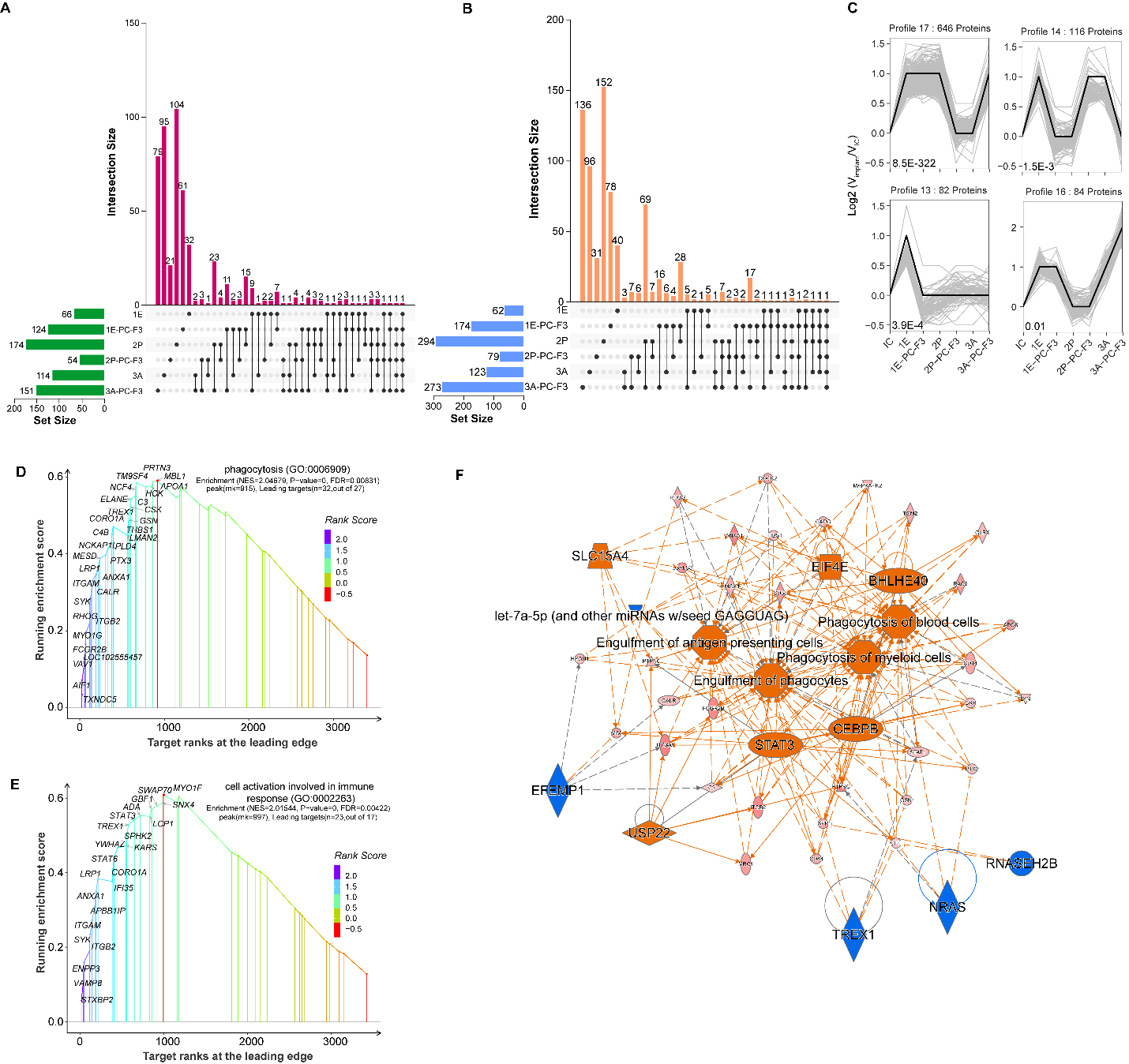


Fig. S2. Protein analysis results for acute phase.


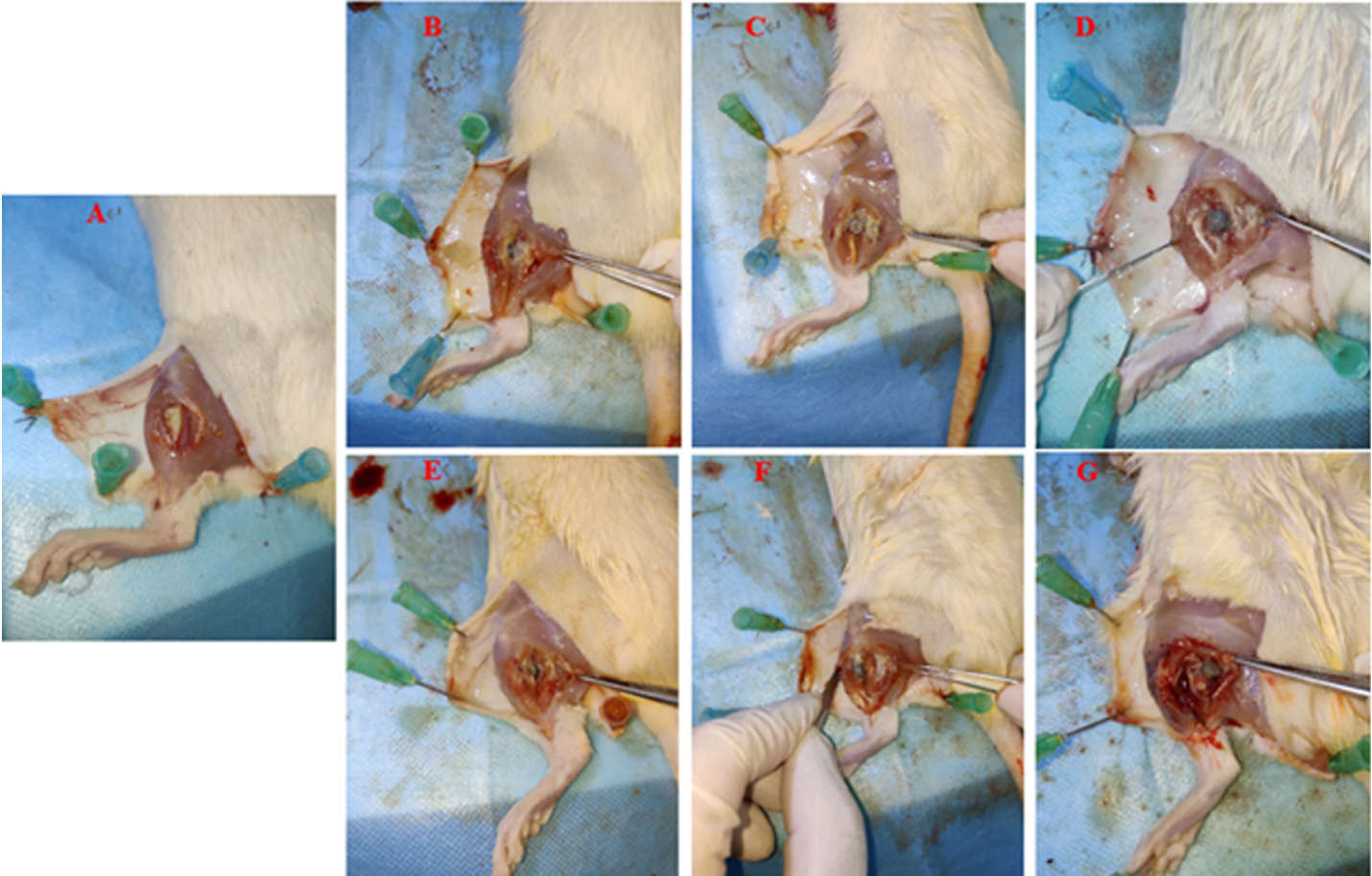


Fig. S3. Morphology of implantation sites.


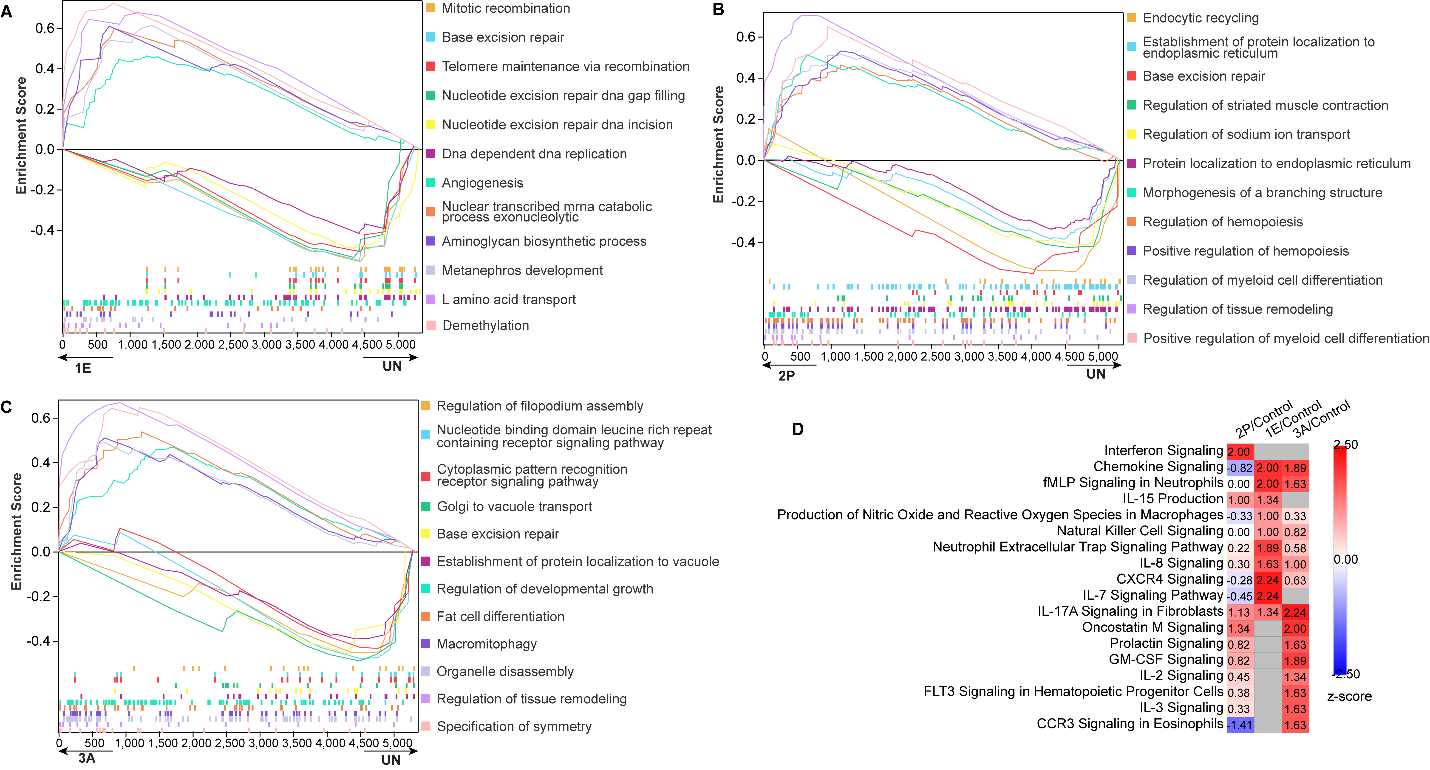


Fig. S4. Osteoblast proteomic analyses.


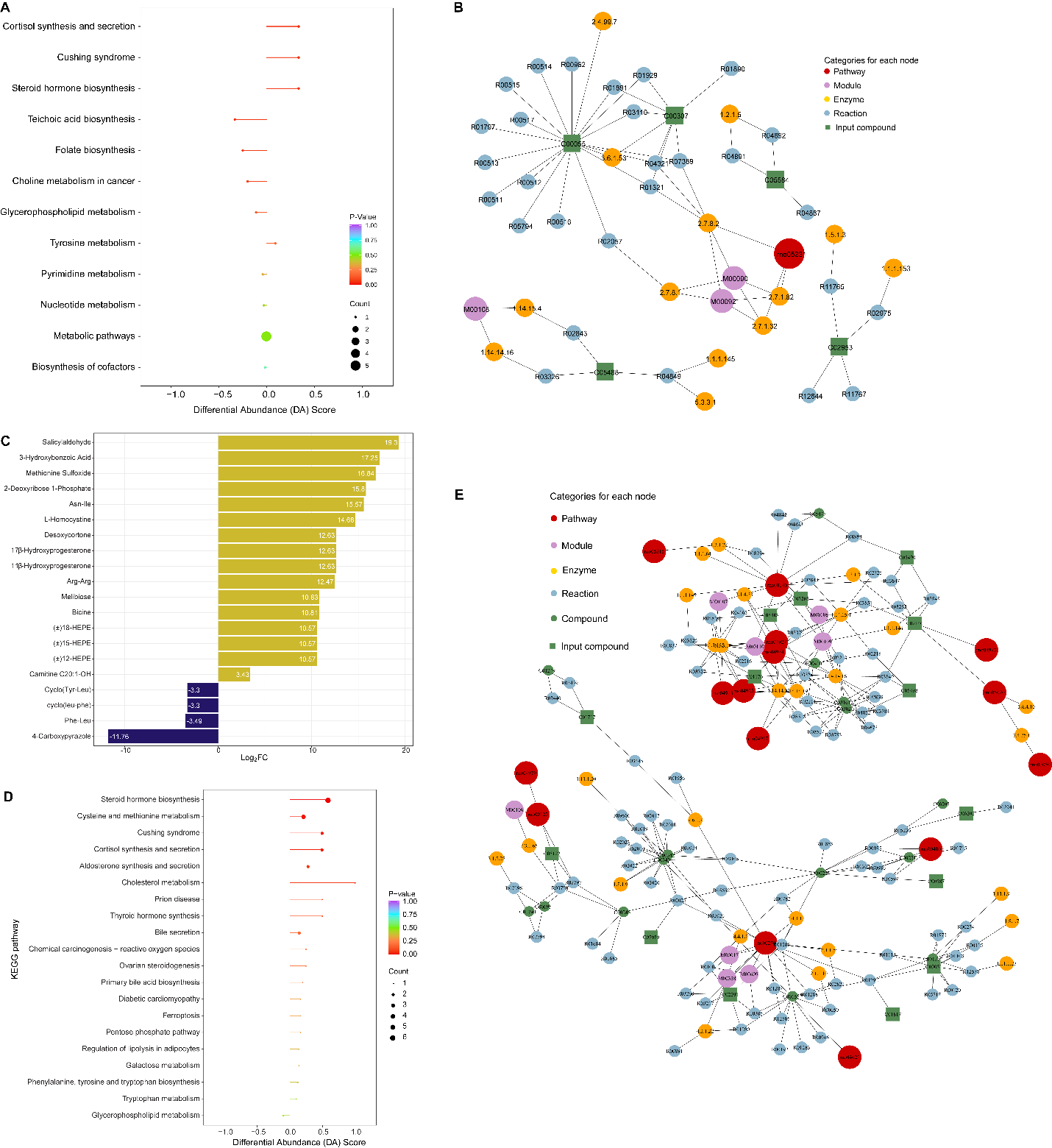


Fig. S5. Combined metabolomics analyses.
