# Supplementary material for: Degradable Magnesium Implants with Caerin 1.9-Polycaprolactone Coatings Provide Extended Antibacterial Resistance and Outstanding Biocompatibility
Source: Biomater Res. 2025 Oct 28;29:0257. doi: 10.34133/bmr.0257 (PMC12559799; doi:10.34133/bmr.0257)
Supplement: Supplementary 1 — Figs. S1 to S5 Tables S1 to S6 [file bmr.0257.f1.zip › Table S1 Ordinary one-way ANVOA with post doc test.docx]

**Results of Ordinary one-way ANOVA test**

**TNF-a**

| **ANOVA summary** |  |
| --- | --- |
| F | 2.171 |
| P value | 0.1953 |
| P value summary | ns |
| Significant diff. among means (P < 0.05)? | No |
| R square | 0.4198 |
|  |  |
| **Brown-Forsythe test** |  |
| F (DFn, DFd) | 0.1960 (2, 6) |
| P value | 0.8270 |
| P value summary | ns |
| Are SDs significantly different (P < 0.05)? | No |

**IL-1β/IL-1F2**

| **ANOVA summary** |  |
| --- | --- |
| F | 17.58 |
| P value | 0.0031 |
| P value summary | ** |
| Significant diff. among means (P < 0.05)? | Yes |
| R square | 0.8542 |
|  |  |
| **Brown-Forsythe test** |  |
| F (DFn, DFd) | 1.010 (2, 6) |
| P value | 0.4188 |
| P value summary | ns |
| Are SDs significantly different (P < 0.05)? | No |

**IL-10**

| **ANOVA summary** |  |
| --- | --- |
| F | 0.9623 |
| P value | 0.4340 |
| P value summary | ns |
| Significant diff. among means (P < 0.05)? | No |
| R square | 0.2429 |
|  |  |
| **Brown-Forsythe test** |  |
| F (DFn, DFd) | 0.9571 (2, 6) |
| P value | 0.4358 |
| P value summary | ns |
| Are SDs significantly different (P < 0.05)? | No |

**Results of Tukey’s Post-hoc tests**

**TNF-a**

| Tukey's multiple comparisons test | Mean Diff. | 95.00% CI of diff. | Significant? | Summary | Adjusted P Value |  |
| --- | --- | --- | --- | --- | --- | --- |
| Control vs. 3A | -0.02413 | -0.06581 to 0.01754 | No | ns | 0.2550 | A-B |
| Control vs. 3APCF3 | -0.02487 | -0.06654 to 0.01681 | No | ns | 0.2385 | A-C |
| 3A vs. 3APCF3 | -0.0007333 | -0.04241 to 0.04094 | No | ns | 0.9984 | B-C |

**IL-1β/IL-1F2**

| Tukey's multiple comparisons test | Mean Diff. | 95.00% CI of diff. | Significant? | Summary | Adjusted P Value |  |
| --- | --- | --- | --- | --- | --- | --- |
| Control vs. 3A | -0.06303 | -0.1071 to -0.01898 | Yes | * | 0.0109 | A-B |
| Control vs. 3APCF3 | -0.08107 | -0.1251 to -0.03702 | Yes | ** | 0.0032 | A-C |
| 3A vs. 3APCF3 | -0.01803 | -0.06208 to 0.02602 | No | ns | 0.4669 | B-C |

**IL-10**

| Tukey's multiple comparisons test | Mean Diff. | 95.00% CI of diff. | Significant? | Summary | Adjusted P Value |  |
| --- | --- | --- | --- | --- | --- | --- |
| Control vs. 3A | 0.01163 | -0.01508 to 0.03835 | No | ns | 0.4281 | A-B |
| Control vs. 3APCF3 | 0.003000 | -0.02372 to 0.02972 | No | ns | 0.9373 | A-C |
| 3A vs. 3APCF3 | -0.008633 | -0.03535 to 0.01808 | No | ns | 0.6082 | B-C |
