# Supplementary material for: Degradable Magnesium Implants with Caerin 1.9-Polycaprolactone Coatings Provide Extended Antibacterial Resistance and Outstanding Biocompatibility
Source: Biomater Res. 2025 Oct 28;29:0257. doi: 10.34133/bmr.0257 (PMC12559799; doi:10.34133/bmr.0257)
Supplement: Supplementary 1 — Figs. S1 to S5 Tables S1 to S6 [file bmr.0257.f1.zip › Table S2 Nucleotide sequences of quantitative PCR.docx]

Table S1. Nucleotide sequences of the primers used for quantitative PCR analysis

| Primer | Primer sequence (5′−3′) |
| --- | --- |
| β-actin | Forward TATAAAACCCGGCGGCGCA |
|  | Reverse GTCATCCATGGCGAACTGGTG |
| BRF1_1 | Forward CCACTCTTTCCCCAAGAGAAT |
|  | Reverse GAGGAACAGAACTGTGTTTTGATGT |
| BRF1_2 | Forward ATGGTGGGACGAGGATACCTA |
|  | Reverse GCTGCAAATTCTCTTGGGGAA |
| Ctnna1 | Forward CAGTTCGCTGCAGAAATGAC |
|  | Reverse CCTGTGTAACAAGAGGCTCCA |
| KAT6A | Forward ATGGTAAAACTCGCTAACCCG |
|  | Reverse CGTCCCGTCTTTGACGCTC |
